# Longitudinal dynamic single-cell mass cytometry analysis of peripheral blood mononuclear cells in COVID-19 patients within 6 months after viral RNA clearance

**DOI:** 10.1186/s12879-024-09464-0

**Published:** 2024-06-06

**Authors:** Diwenxin Zhou, Shuai Zhao, Keting He, Qiuhong Liu, Fen Zhang, Zhangya Pu, Lanlan Xiao, Lingjian Zhang, Shangci Chen, Xiaohan Qian, Xiaoxin Wu, Yangfan Shen, Ling Yu, Huafen Zhang, Jiandi Jin, Min Xu, Xiaoyan Wang, Danhua Zhu, Zhongyang Xie, Xiaowei Xu

**Affiliations:** State Key Laboratory for Diagnosis and Treatment of Infectious Diseases, National Clinical Research Center for Infectious Diseases, National Medical Center for Infectious Diseases, Collaborative Innovation Center for Diagnosis and Treatment of Infectious Diseases, The First Affiliated Hospital, Zhejiang University School of Medicine, 79 Qingchun Rd., Hangzhou City, 310003 China

**Keywords:** COVID-19, Dynamics, PBMC (peripheral blood mononuclear cells), Recovery, CyTOF

## Abstract

This study investigates the longitudinal dynamic changes in immune cells in COVID-19 patients over an extended period after recovery, as well as the interplay between immune cells and antibodies. Leveraging single-cell mass spectrometry, we selected six COVID-19 patients and four healthy controls, dissecting the evolving landscape within six months post-viral RNA clearance, alongside the levels of anti-spike protein antibodies. The T cell immunophenotype ascertained via single-cell mass spectrometry underwent validation through flow cytometry in 37 samples. Our findings illuminate that CD8 + T cells, gamma-delta (gd) T cells, and NK cells witnessed an increase, in contrast to the reduction observed in monocytes, B cells, and double-negative T (DNT) cells over time. The proportion of monocytes remained significantly elevated in COVID-19 patients compared to controls even after six-month. Subpopulation-wise, an upsurge manifested within various T effector memory subsets, CD45RA + T effector memory, gdT, and NK cells, whereas declines marked the populations of DNT, naive and memory B cells, and classical as well as non-classical monocytes. Noteworthy associations surfaced between DNT, gdT, CD4 + T, NK cells, and the anti-S antibody titer. This study reveals the changes in peripheral blood mononuclear cells of COVID-19 patients within 6 months after viral RNA clearance and sheds light on the interactions between immune cells and antibodies. The findings from this research contribute to a better understanding of immune transformations during the recovery from COVID-19 and offer guidance for protective measures against reinfection in the context of viral variants.

**Supplementary Information:**

The online version contains supplementary material available at 10.1186/s12879-024-09464-0.

## Introduction

The global COVID-19 pandemic, which was caused by the severe acute respiratory syndrome coronavirus 2 (SARS-CoV-2) has left an indelible mark, it poses a grave threat to both public health and daily routines, leading to a substantial surge in infections and fatalities on a global scale [1]. Since the discovery of SARS-CoV-2, the number of patients experiencing reinfection has been continuously rising. There is evidence to suggest that reinfection is associated with an increase in risks of death and hospitalization during the acute phase and after, as well as the occurrence of multiple organ sequelae [2]. SARS-CoV-2 targets cells through its structural spike (S) protein, which binds with the angiotensin-converting enzyme 2 (ACE2) receptor [3]. The resulting infection sets off both innate and adaptive immune responses, disrupts lymphopoiesis, and amplifies lymphocyte apoptosis. In severe instances, an excessive immune reaction can trigger a “cytokine storm” [4], activating the coagulation cascade and depleting clotting factors [5].

Once the immune system triumphs over the virus, the body begins a series of immune reconstitution and recovery processes. Within the initial 14 days following viral RNA clearance, peripheral blood mononuclear cells (PBMCs) exhibit notable alterations in the proportions of monocytes, CD4 + T cells, and CD8 + T cells [6]. However, the longitudinal dynamics of immune cells in recovering patients and the relationship between immune cells and antibodies remain uncertain. It remains unclear whether persistent immune alterations contribute to lingering symptoms.

In this investigation, we utilized single-cell time-of-flight mass spectrometry (CyTOF) to comprehensively scrutinize the dynamics occurring within PBMCs from six COVID-19 patients during the six months following viral RNA clearance. We validated the dynamic changes of T cells in additional 37 samples. Our study also encompassed an examination of anti-S antibodies (including both IgM and IgG), to decipher the immune changes manifest in convalescent patients as well as the interplay between immune cell subpopulations and antibodies. Throughout our study, several distinct shifts within PBMCs came to light, potentially augmenting our understanding of immune change during the recovery phase post COVID-19. This contributes to formulating strategies for preventing reinfections, thereby reducing mortality and complications caused by SARS-CoV-2.

## Methods

### Patients

All patients admitted to the First Affiliated Hospital, Zhejiang University School of Medicine with SARS-CoV-2 infection, confirmed by testing respiratory specimens by real-time reverse transcription–polymerase chain reaction (RT-PCR) (Shanghai Bio-Germ Medical Technology Co., Ltd., Shanghai, China) were enrolled in this study along with four healthy controls (HCs) from the hospital health management center. None of enrolled patients and HCs received vaccine injection. The study was approved by the Research Ethics Committee of the First Affiliated Hospital, Zhejiang University School of Medicine, and all subjects provided written informed consent.

Disease severity was classified according to the Diagnostic and Treatment Protocol for COVID-19 in China (5th edition). Severe disease was defined as one of the following manifestations: respiratory rate ≥ 30 breaths/min; oxygen saturation ≤ 93% at rest; arterial partial pressure of oxygen/fraction of inspired oxygen ratio ≤ 300 mm Hg; progression (> 50%) in lung imaging lesions within 24–48 h; or admission to the ICU.

### Sample collection

Six COVID-19 patients and four HCs’ whole blood samples were collected at 1 week (T1), 3 months (T2), and 6 months (T3) after the first negative result for SARS-CoV-2 RNA by RT-PCR for CyTOF analysis. Other twenty-eight COVID-19 patients were enrolled for cytometry after the first negative result for SARS-CoV-2 RNA by RT-PCR, twenty whole blood samples were collected at 3 months (T2), and seventeen whole blood samples were collected at 6 months (T3). All the samples were collected from February 2020 to September 2020. PBMCs were separated by Ficoll density gradient centrifugation and cryopreserved in 90% fetal calf serum with 10% DMSO in liquid nitrogen. Serum samples were stored at − 80 °C in an ultra-low temperature freezer for further analysis.

### CyTOF

Detailed experimental procedures and data analysis for cyTOF are listed in Supplementary Materials. Briefly, PBMCs were resuspended in the cell staining (1× PBS + 0.5% bovine serum albumin) buffer, and a total of 31 cell-surface antibodies (listed in Supplementary Table 1) labeling with the indicated metal tag were used to identify the subpopulations of PBMCs. Cells were incubated in Fc receptor blocking solution and then fixed in 200 µL intercalation solution (Maxpar Fix and Perm Buffer containing 250nM 191/193Ir, Fluidigm) overnight. After fixation, cells were washed once with FACS buffer (1×PBS + 0.5% bovine serum albumin) and then permeabilization buffer (Scientific) and stained with intracellular antibody cocktail for 30 min on ice. Cells were washed and resuspended in deionized water, followed by addition of 20% EQ beads (Fluidigm), and then evaluated by mass cytometry (Helios, Fluidigm). Manually gate data using a FlowJo software to exclude debris, dead cells and doublets, leaving live, single immune cells (Becton Dickinson), gating strategy was shown in Supplementary Fig. 1.

### T cell immunophenotype by polychromatic flow cytometry

After thawing PBMCs, we washed PBMCs with RPMI 1640 supplemented with 10% fetal bovine serum. After the secondary washing with PBS, at least 1,000,000 PBMCs were counted and stained with viability dye and seven fluorescent monoclonal antibodies (BD Pharmingen) for surface antigens: CD45RA-FITC, CD3-APC-H7, CD4-BV510, CD8-APC, CCR7-BV421. A minimum of 50,000 cells per sample were collected with the use of a CytoFLEX LX flow cytometer (Beckman Coulter). Supplementary Table 2 lists all antibodies used in this study. Cellular phenotypes were analyzed with the use of FlowJo software (Becton Dickinson).

### Antibody detection

Serum antibody levels in COVID-19 patients were detected using a new coronavirus IgG/IgM dual detection kit using quantum dot fluorescence immunochromatography. The reagent strip was pre-coated with anti-human IgM (MRM01; Ebiocore Co., Hangzhou, China), anti-human IgG (ABSKR111; Winbio Biotechnology Co., Xiamen, China), and goat anti-rabbit IgG in the detection line area and quality control line area, and S1 protein (DRA38; Novoprotein, Shanghai, China) fluorescently labeled with quantum dots was coated in the release pad area. Aliquots of 5 µL of serum samples were diluted 1:100 with loading buffer (0.02 M PBS + 0.1% BSA + 0.125% Tween-20 + 0.15% proclin-300, pH 7.4) for testing. An aliquot of 100 µL of the diluted sample was pipetted into the sample hole of the reagent strip. After reaction for 10 min, the fluorescence intensity was measured using the instrument for quantitative analysis. The cutoff value for seropositivity was set as follows: the average value at optical density 450 (1:50 dilution) was tested in 16,100 negative serum samples; for three-fold average > 0.1, the cutoff value was calculated as the three-fold average; for 3-fold average < 0.1, the cutoff value was set to 0.1. In the present study, the IgM and IgG cutoff values were 0.1 and 0.2, respectively.

### Statistical analysis

Statistical analyses were performed using SPSS (ver. 22.0; SPSS Inc., Chicago, IL, USA) and Prism 9.0 (GraphPad Software Inc, la Jolla, USA). Continuous variables are expressed as the mean ± SD and categorical variables as the number [7]. The paired Student’s *t* test, unpaired *t* test or non-parametric Mann–Whitney U test was used for analysis of continuous data, as appropriate. Categorical data were compared using the χ^2^ test. Spearman’s rank correlation coefficient was used to evaluate the linear correlations between variables. In all analyses, *P* < 0.05 was taken to indicate statistical significance.

## Results

### Demographic and clinical characteristics

A total of thirty-four COVID-19 patients and four healthy controls (HCs) were included in this study. Six patients and four healthy controls were included in cyTOF analysis, and another twenty-eight patients were included in flow cytometry analysis. Supplementary Tables 3 and 4 present the demographic and clinical characteristics at admission. The principal clinical features of patients at different time points are outlined in Tables 1 and 2. In comparison to the T1 group, T2 and T3 groups exhibited notably elevated levels of hemoglobin and albumin, while the lactate dehydrogenase (LDH) level saw a significant reduction in the T2 and T3 groups. As time progressed, the concentrations of anti-S IgG and IgM exhibited a decline, with the IgM titer showing a substantial decrease at the 6-month mark in comparison to the measurement taken 7 days after viral RNA clearance.

**Table 1:** clinical parameters of six COVID-19 patients analysed by CyTOF on different time point

	Within 7 days after RNA shedding	3 months after RNA shedding	6 months after RNA shedding
	T1	T2	T3
White blood counts (*10^9^/L)	7.4 ± 2.5	5.5 ± 1.6	5.4 ± 1.5
Leukocytes (*10^9^/L)	5.4 ± 2.6	3.1 ± 1.2	3 ± 0.8
Lymphocytes (*10^9^/L)	1.4 ± 0.7	1.8 ± 0.4	1.8 ± 0.5
Hemoglobin (g/L)	127.2 ± 9.3	147.5 ± 13.9*	148 ± 13.2*
Platelets (*10^9^/L)	199 ± 99.5	218.3 ± 67.7	217.8 ± 62.9
Albumin (g/L)	41.8 ± 4.7	49.6 ± 1.2**	47.4 ± 1.4*#
ALT (U/L)	38.8 ± 24.4	22 ± 8.2	23.7 ± 13.7
AST (U/L)	22.8 ± 9.4	20.5 ± 4	20.3 ± 12.2
Creatine (µmol/L)	60.8 ± 12.1	70.3 ± 12.6	73.5 ± 11.2
LDH (U/L)	251.5 ± 41.2	208.2 ± 16.6*	209 ± 22.5*
Anti-S IgG	2.378 ± 2.861	1.948 ± 3.152	1.436 ± 2.97*#
Anti-S IgM	0.463 ± 0.454	0.179 ± 0.165	0.047 ± 0.042*#

*Abbreviation* ALT, Alanine transaminase; AST, Aspartate Aminotransferase; LDH, lactate dehydrogenase. *: *p* < 0.05 compare to T1 group; ** *p* < 0.01 compare to T1 group; # *p* < 0.05 compare to T2 group

**Table 2:** clinical parameters of twenty-eight COVID-19 patients analysed by flow cytometry on different time point

	3 months after RNA shedding	6 months after RNA shedding
	T2	T3
White blood counts (*10^9^/L)	8.1 ± 5.4	8.0 ± 5.4
Leukocytes (*10^9^/L)	79.8 ± 12.6	76.8 ± 13.8
Lymphocytes (*10^9^/L)	13.4 ± 9.0	15.3 ± 9.8
Hemoglobin (g/L)	138.5 ± 12.8	143.8 ± 14.0
Platelets (*10^9^/L)	180.7 ± 43.1	181.6 ± 66.2
INR	1.0 ± 0.1	1.0 ± 0.1
ALT (U/L)	23.4 ± 11.9	24.8 ± 12.5
AST (U/L)	23.0 ± 8.8	24.9 ± 9.5
Albumin(g/dL)	37.6 ± 6.1	39.1 ± 5.4
Creatine (µmol/L)	74.9 ± 17.7	74.4 ± 18.0
C-creative protein(mg/L)	23.4 ± 31.1	24.8 ± 38.0
LDH (U/L)	249.5 ± 62.7	260.0 ± 92.3

*Abbreviation* ALT, Alanine transaminase; AST, Aspartate Aminotransferase; LDH, lactate dehydrogenase

### t-SNE, clustering analysis, and main cell surface marker expression analysis by CyTOF

**Fig. 1:**
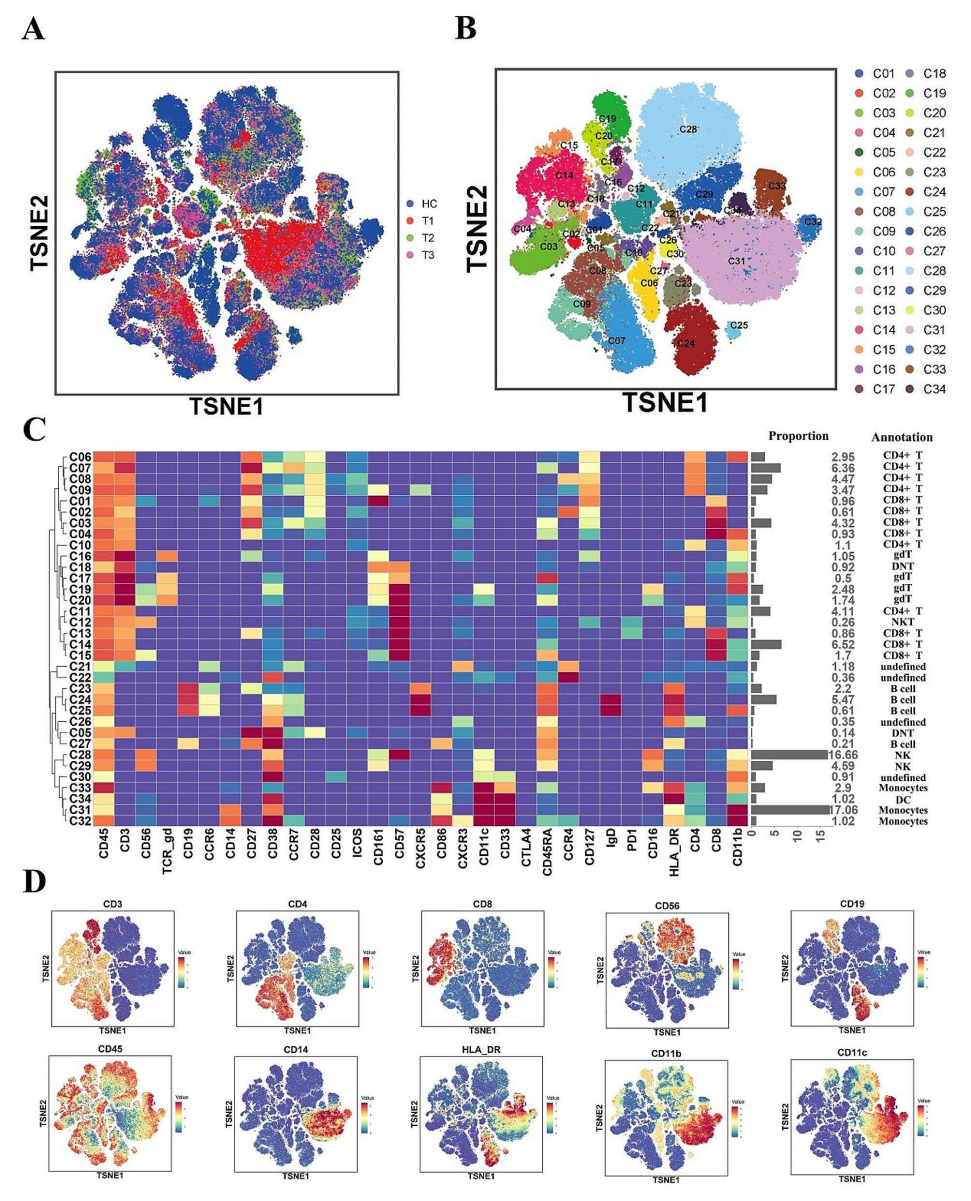
Overview of the cyTOF landscape of the PBMCs of patients recovered from COVID-19. (**A**) t-SNE map colored by different groups. (**B**) t-SNE map colored by different cell subsets. (**C**) clustering map of cell surface markers expressed by different cell subsets. (**D**) t-SNE map of the expression of key cell surface markers

Six COVID-19 patients and four healthy control subjects (HCS) were subjected to detection and projection onto a two-dimensional plane using t-SNE (Fig. 1A), while the t-SNE projections of the T1, T2, T3, and HC groups can be observed in Supplementary Fig. 2A. The spatial representations of cells in different groups revealed several distinct features, especially in the T1 group. Subsequently, we classified different cell types based on the distinct expression of characteristic surface markers. By clustering total PBMCs across all 22 samples, 34 distinct subpopulations were identified and categorized within the t-SNE projection (Fig. 1B). The classification criteria for different groups in t-SNE are detailed in Supplementary Fig. 2B. By considering both Fig. 1A and B, discernible disparities in the distributions of several subpopulations (C28, C29, C31, etc.) between the T1 group and other groups suggested quantitative and functional shifts within corresponding immune cells during the recovery process post COVID-19. The clustering map, specific subpopulation designations, and their respective proportions are delineated in Fig. 1C. Notably, CD4 + T cells, CD8 + T cells, natural killer (NK) cells, and monocytes constituted the predominant population. The distributions of markers that define key subpopulations are illustrated in Fig. 1D.

In general, the classification of cell subsets is reflected in this section, which lays the foundation for the subsequent analysis of immune cells changes in six months after viral RNA clearance.

### Dynamic changes in major cell types

**Fig. 2:**
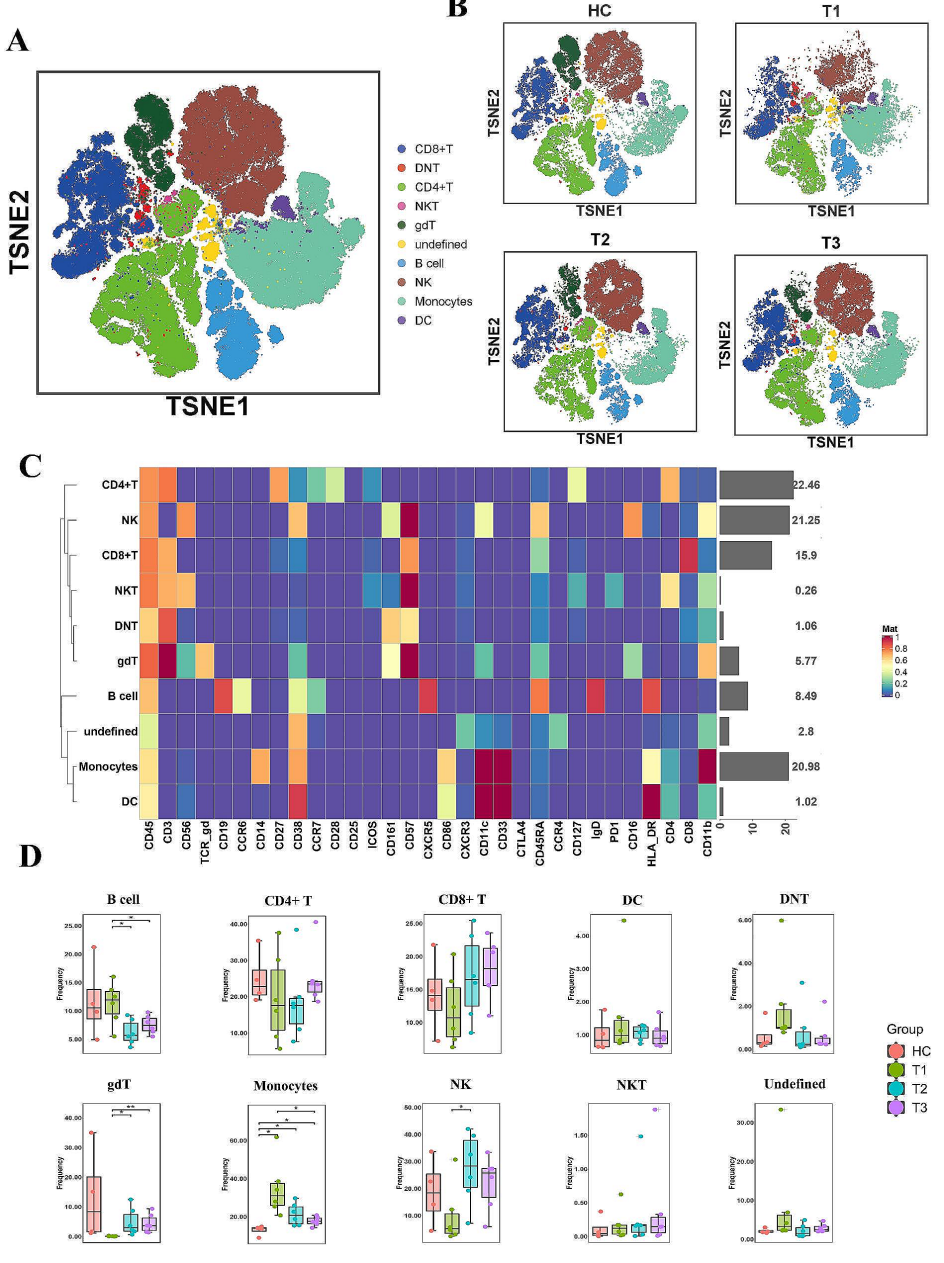
Major cell types analysis of different groups in patients recovered from COVID-19. (**A**) t-SNE map colored by major cell types. (**B**) t-SNE map colored by major cell types in different groups. (**C**) clustering map of cell surface markers expressed by different cell subsets. (**D**) comparison of the cells ratio in different groups using non-parametric Mann–Whitney U test

We classified the 34 distinct cell subgroups into 10 major cell types (Fig. 1C), and the respective proportions of these cell groups are itemized in Supplementary Table 5. The t-SNE projections showcasing these 10 major cell groups are demonstrated in Fig. 2A and B, while the clustering map detailing differential expression of.

surface markers can be found in Fig. 2C. Among these major cell groups, the ones that exhibited differential distributions between the T1 group and the other three groups encompassed NK cells, monocytes, and gdT cells. Subsequently, we conducted a non-parametric Mann-Whitney U test to compare cell proportions across the HC, T1, T2, and T3 groups (Fig. 2D). The T2 group registered significantly higher proportions of NK and gdT cells in contrast to the T1 group, whereas B cells and monocytes experienced noteworthy reductions in the T2 and T3 groups compared to the T1 group. In relation to the HC group, the T1 group displayed a considerable increase in the monocyte population, while the T2 and T3 groups demonstrated a slight decrease, yet the monocyte population remained notably elevated in the T3 group.

**Fig. 3:**
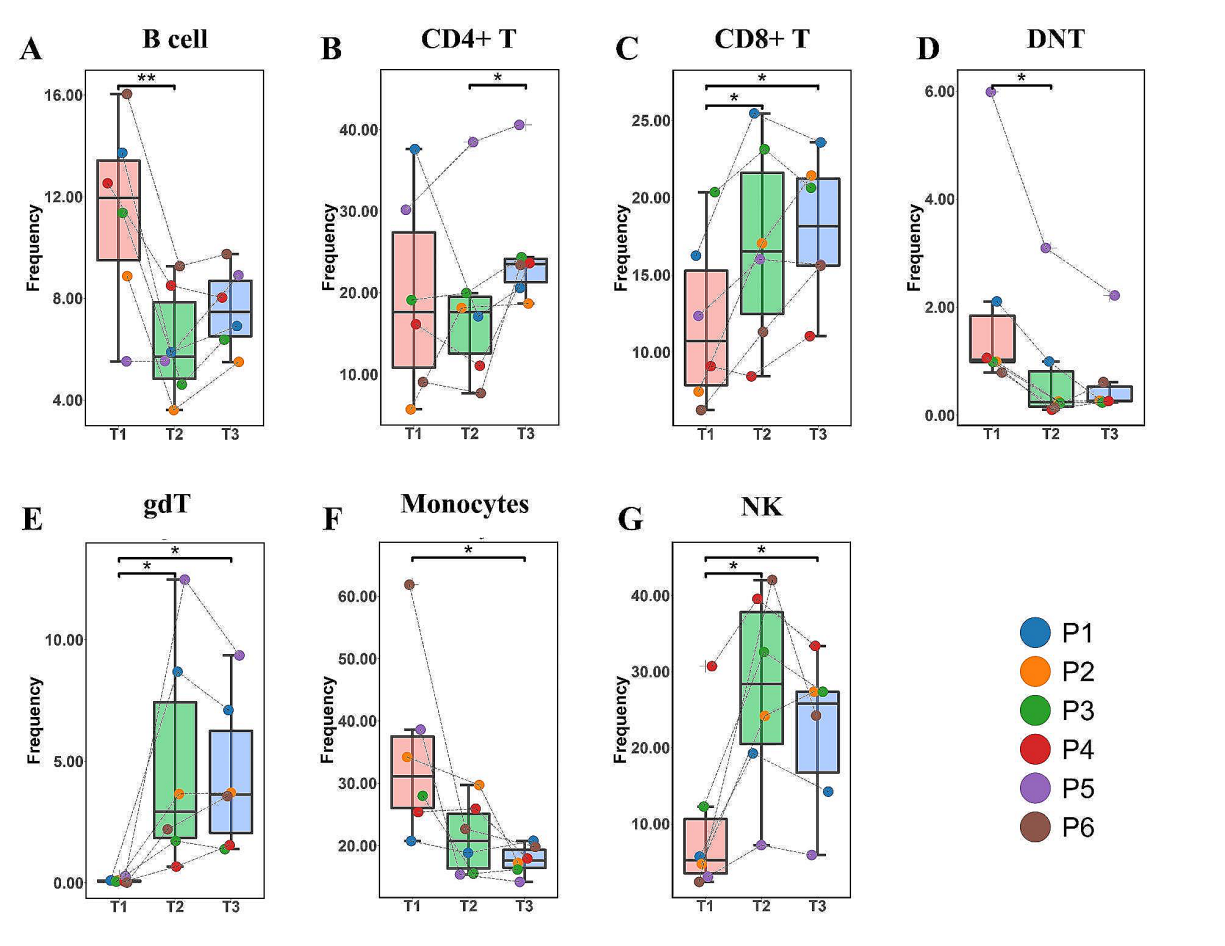
Dynamic changes of major cell types in COVID-19 patients. The dynamic changes of ratio of B cell (**A**), CD4 + T cell (**B**), CD8 + T cell (**C**), DNT cell (**D**), gdT cell (**E**), monocytes (**F**) and NK cell (**G**) were shown. *: *p* < 0.05

To delve into the trends of various cell groups following viral RNA clearance, we performed a paired Student’s t-test to compare changes in their proportions among the T1, T2, and T3 groups. As shown in Fig. 3, the T2 and T3 groups exhibited heightened proportions of CD8 + T cells, gdT cells, and NK cells in comparison to the T1 group, whereas monocytes, B cells, and double-negative T (DNT) cells displayed diminishing proportions over time.

Genernally, this section shows t-SNE projections and the clustering map of ten major cell populations, and their changes in six months after infection. NK cells, monocytes, gdT cells, B cells, CD8 + T cells and DNT cells have significant differences between groups using different analysis methods, these cells should be paid more attention in subsequent research.

### Dynamic changes in different types of T cells

Subsequently, an analysis was conducted on the alterations in all 34 subpopulations across the diverse groups. A comparison was executed among the four groups using the non-parametric Mann-Whitney U test, and the subgroups with significant differences are illustrated in Supplementary Fig. 3. Notable variations were noted in C16 and C31 between the HCs and the T1 group.

**Fig. 4:**
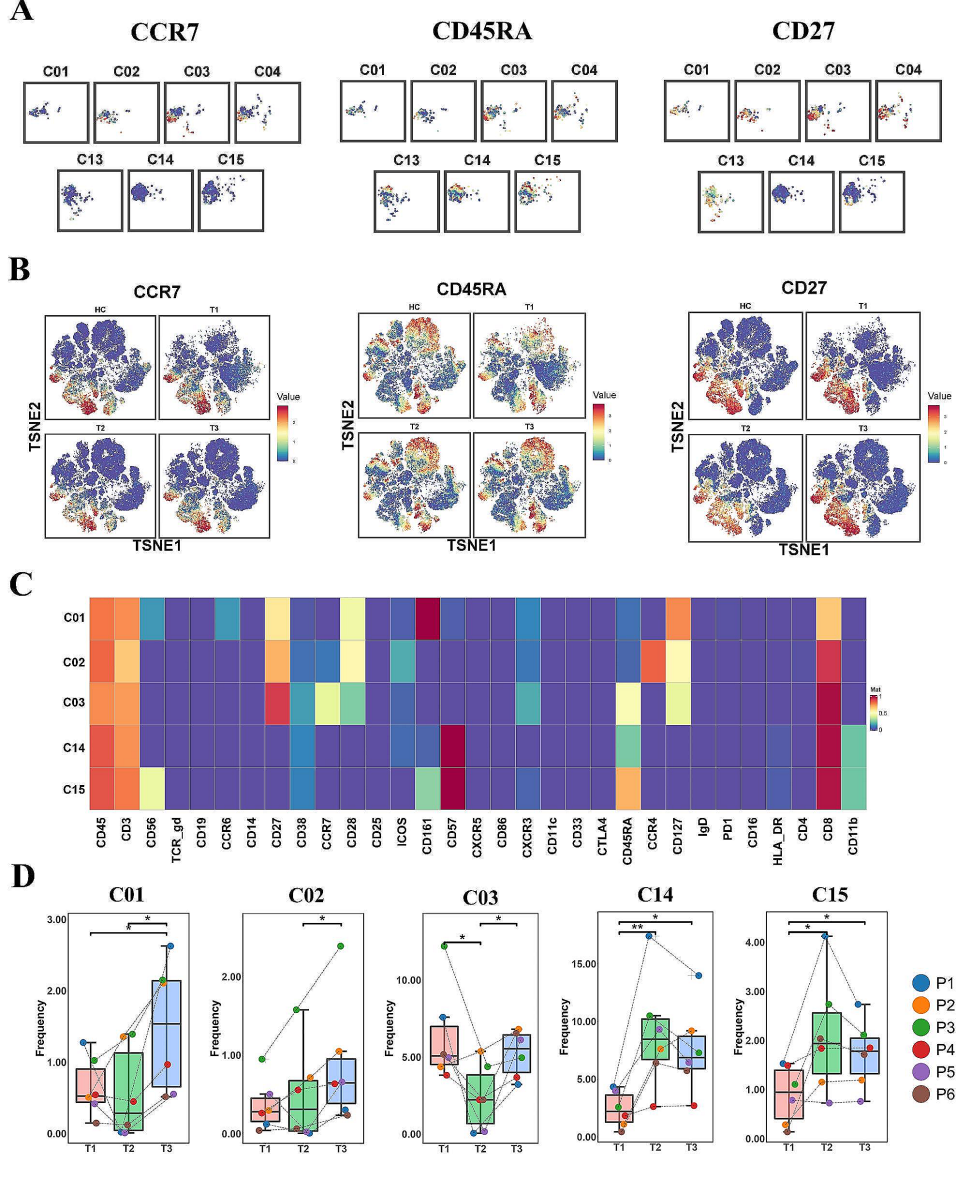
Dynamic changes of CD8^+^ T cells of different groups. Distributions of CCR7, CD45RA and CD27 on the subpopulations of CD8 + T cells and all cells in the t-SNE map were shown in (**A**) and (**B**). (**C**) Clustering of significantly changed subpopulations of CD8 + T cells. (**D**) The dynamic changes of ratio of subpopulations of CD8^+^ T cells. *: *p* < 0.05; **: *p* < 0.01

The investigation then shifted to the changes within the CD8 + T cells. Employing the combination of CD45RA, CD27, and CCR7 surface markers, a total of seven CD8 + T cell subpopulations were identified: naïve cells (CD45RA + CD27 + CCR7+ [C03, C04]), T effector memory (TEM) cells (CD45RA − CD27 + CCR7− [C01, C02, C13]), and CD45RA + T effector memory (TEMRA) cells (CD45RA + CD27 − CCR7− [C14, C15]). The expressions of CCR7, CD45RA, and CD27 in various CD8 + T cell subpopulations and different groups are demonstrated in Fig. 4A and B. Among.

these subpopulations, five (C01, C02, C03, C14, and C15) exhibited significant changes throughout the recovery period. The heatmap illustrating cell surface marker expression for these five subpopulations is depicted in Fig. 4C. Increases were observed in the populations of TEM cells (C01 and C02) and TEMRA cells (C14 and C15), while the naïve T cell population (C03) demonstrated a decrease within 3 months after viral RNA clearance, followed by an increase at 6 months (Fig. 4D).

**Fig. 5:**
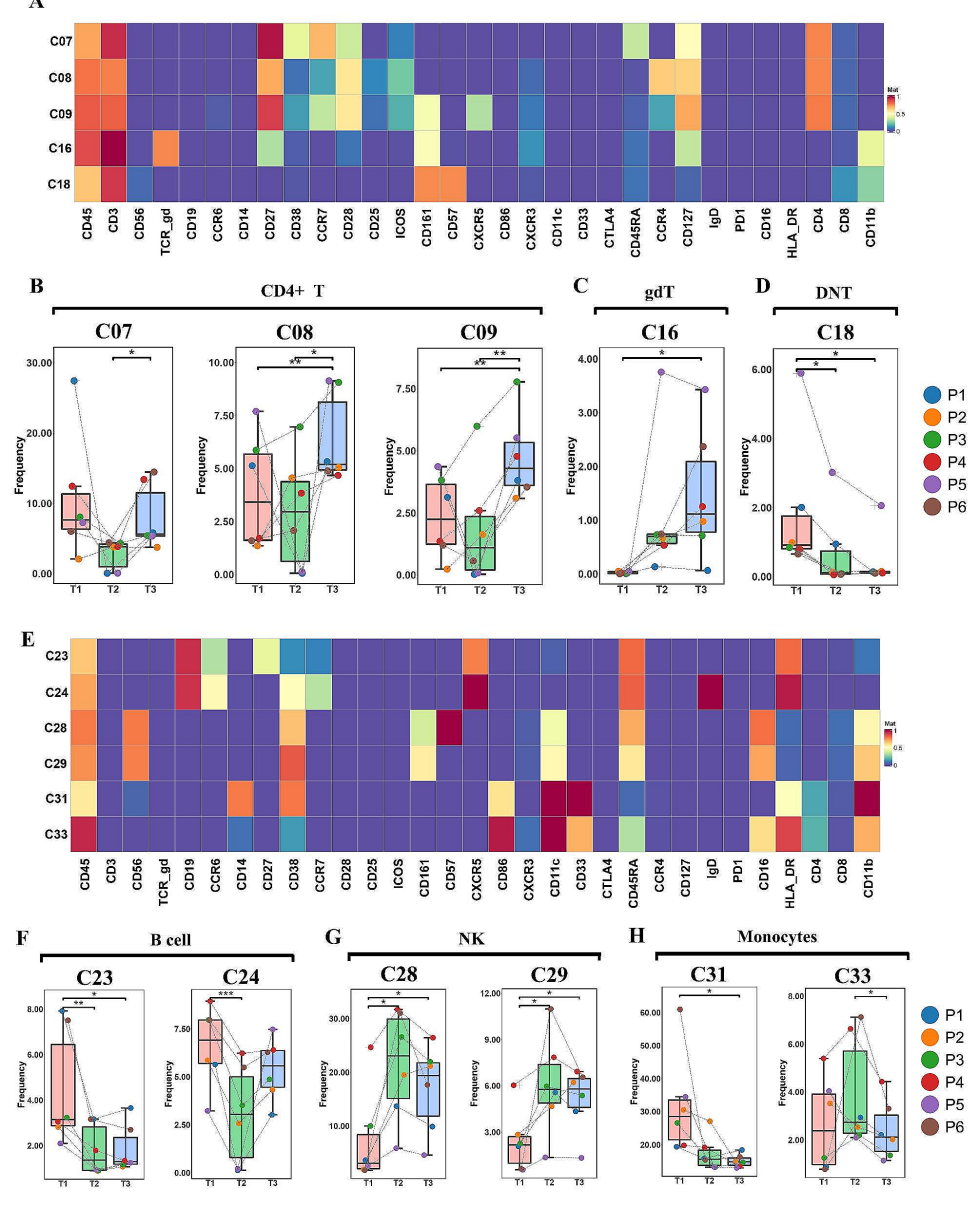
Dynamic changes of CD4 + T, gdT, DNT, B cells, NK and monocytes. Clustering of significantly changed subpopulations of CD4 + T, gdT and DNT cells is shown in (**A**). The dynamic changes of ratio of subpopulations of CD8^+^ T cells (**B**), gdT (**C**), and DNT (**D**) cells. (**E**) Clustering of significantly changed subpopulations of B cells, NK cells and Monocytes. The dynamic changes of ratio of subpopulations of B cells (B), NK cells (**C**), and Monocytes (**D**)

Similarly, an analysis of CD4 + T cells was conducted. Six CD4 + T cell subsets were defined, mirroring the categories described earlier for CD8 + T cells, encompassing naïve T cells (C07), T central memory (TCM) cells (CD45RA − CD27 + CCR7+, C09), and TEM cells (CD45RA − CD27 + CCR7−, [C06, C08], CD45RA − CD27 − CCR7−, [C10, C11]). Three of these subpopulations (C07, C08, and C09) displayed significant changes, and their surface marker expression densities are depicted in Fig. 5A. These three groups showed a declining trend at 3 months after viral RNA clearance, but experienced a significant increase at the 6-month mark (Fig. 5B).

To validate the immune system reconfiguration and the dynamic alterations of T cell subsets in COVID-19 patients following recovery, we selected 20 patients who had recovered from COVID-19 for 3 months (referred to as the T2 group mentioned earlier), and 17 patients who had recovered for 6 months (referred to as the T3 group). Recovery was defined as the first negative result for SARS-CoV-2 RNA via RT-PCR. The demographic and clinical characteristics are detailed in Supplementary Table 4. We used flow cytometry to study CD4 + and CD8 + T cells in these patients, focusing on surface markers (CD45RA, CCR7) associated with T cell differentiation. T cells were divided into subgroups, including naive T cells (CCR7 + CD45RA+), central memory T cells (TCM; CCR7 + CD45RA-), effector memory T cells (TEM; CCR7-CD45RA-), and TEMRA (CCR7-CD45RA+), the gating strategy showed in Supplementary Fig. 4, and the proportions of these cell groups are itemized in Supplementary Table 6.

**Fig. 6:**
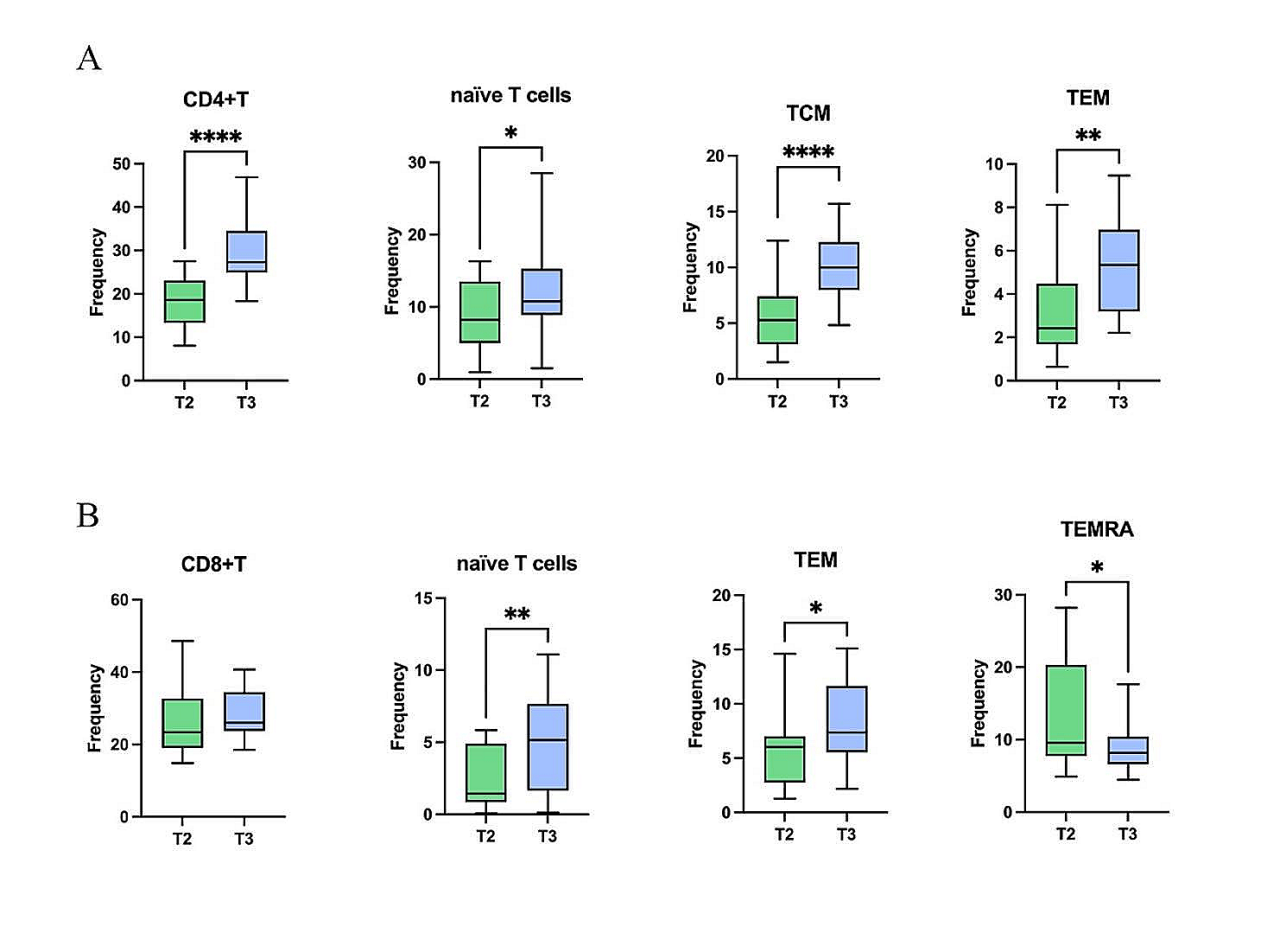
Dynamic changes in T cell subpopulations. The dynamic changes of ratio of subpopulations of CD4 + T cells, CD4 + naive T cells, CD4 + TCM, and CD4 + TEM (**A**). The dynamic changes of ratio of subpopulations of CD8 + T cells, CD8 + naive T cells, CD8 + TEM, and CD8 + TEMRA (**B**).*: *p* < 0.05; **: *p* < 0.01; ***:*p* < 0.001; ****:*p* < 0.0001

We compared the changes in CD4 + T cell proportions (Fig. 6A). The proportion of CD4 + T cells exhibited a significant increase with the extension of viral RNA clearance, eventually approaching levels akin to healthy individuals after 6 months of viral RNA clearance. In CD4 + T cells, the proportions of naive T cells, TCM, and TEM demonstrated an upward trajectory. Notably, the proportions of these cell types were significantly higher after 6 months of viral RNA clearance compared to the measurement taken 3 months after viral RNA clearance. This aligns with the findings derived from the single-cell mass cytometry analysis.

Furthermore, we analyzed the surface markers of CD8 + T cell subsets (Fig. 6B), subsequently comparing the proportions of naive T cells, TEM cells, and TEMRA cells between the T2 and T3 groups. The proportions of naive T cells and TEM cells witnessed a substantial increase at 6 months after viral RNA clearance, in contrast to the proportions recorded 3 months after viral RNA clearance. Conversely, the proportion of TEMRA cells dwindled as the duration of viral RNA clearance increased, which is in harmony with the preceding findings.

Moreover, two subcategories of low-abundance T cells, gdT cells and DNT cells, were delineated. The gdT cells constituted less than 5% of the peripheral lymphocyte population among HCs, fulfilling multifaceted roles such as immune surveillance, immunoregulation, and effector function, all without undergoing clonal expansion [8]. A subset of CD19 + CD161 + CD127 + gdT cells (C16) exhibited a reduction in the T1 group compared to the HC group (Supplementary Fig. 3E), but showed signs of recovery in the T3 group (Fig. 5C). DNT cells represent a minor subset of mature peripheral T cells that might be implicated in systemic inflammation and tissue damage during autoimmune or inflammatory conditions [9]. One such subset, C18, a subpopulation of DNT cells, underwent gradual reduction over the course of the COVID-19 recovery period (Fig. 5D).

In this part, we analyzed the changes of CD8 + T cell, CD4 + T cell, and other low-abundance T cell subsets in T1, T2, and T3 groups. In CD8 + T cells, naïve T cells, TEM cells, TEMRA cells showed significant changes, and in CD4 + T cells, naïve T cells, TCM cells, TEM cells changed sifnificantly, similar results were obtained in flow cytometry validation.

### Dynamic changes in B cells, NK cells, and monocytes

Furthermore, we delved into the alterations within B cell subsets, NK cells, and monocytes, scrutinizing the cell surface marker expression of the subgroups that displayed significant changes (Fig. 5E).

Four distinct B cell subsets (C23, C24, C25, and C27) were identified ( Fig. 1C). Naïve B cells are characterized by CD27-IgD + expression, while memory B cells are identified by CD27 + IgD- expression [10]. The findings unveiled a reduction in both naïve B cells (C23) and memory B cells (C24) during the COVID-19 recovery period (Fig. 5F).

NK cells have been observed to undergo exhaustion during COVID-19 infection [11]. In line with this observation, the proportions of C28 and C29 were notably diminished at the time of viral RNA clearance, but experienced an increase at 3 months and sustained a similar level at 6 months post-viral RNA clearance (Fig. 5G).

Moving forward, we explored the shifts within the monocyte population. Generally, the combination of CD14 and CD16 expression enabled classification of three principal human monocyte subsets: CD14 + CD16 − monocytes, also referred to as “classical” monocytes, and, comprising 10–20%, CD14 + CD16 + intermediate and CD14LowCD16+ “non-classical” monocytes [12]. Our results underscored a decline in the abundance of a subset of classical monocytes (C31) at 6 months after viral RNA clearance (Fig. 5H), although their levels remained higher than those observed in the HC group (Supplementary Fig. 3F). Meanwhile, C33, a subpopulation of non-classical monocytes, exhibited an increase at 3 months, followed by a decline at 6 months (Fig. 5H).

Overall, naïve B cells, memory B cells, NK cells and monocytes had notable alterations in COVID-19 patients within 6 months after viral RNA clearance.

### Correlations between cell subset ratios and antibody concentrations

Recent studies have indicated that the level of anti-SARS-CoV-2 IgM exhibits a gradual decline from after two weeks following the onset of COVID-19, whereas the IgG level experiences a decline within the range of 1 to 2 months [13, 14]. Our findings showed IgG and IgM levels decreased at the 3-month juncture following viral RNA clearance.

**Fig. 7:**
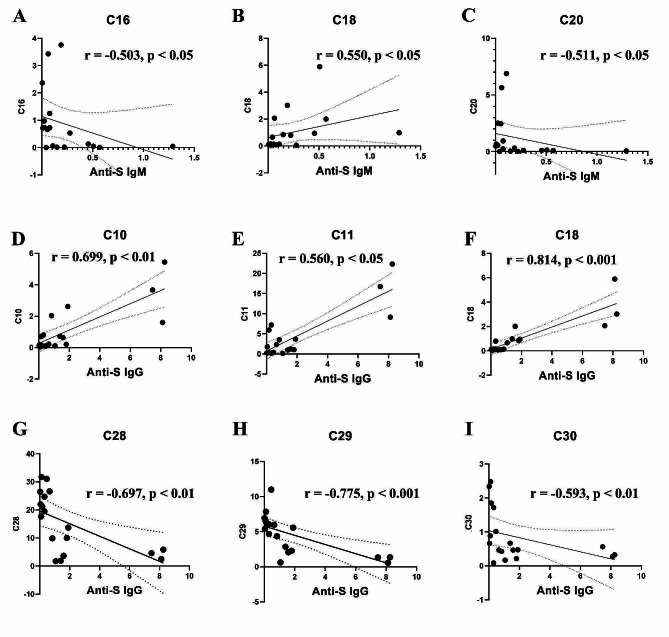
Correlations between the ratio of cell subpopulations and the concentrations of antibodies. Anti-S IgM was negatively correlated to C16 (**A**) and C20 (**C**), but positively correlated to C18 (**B**). Anti-S IgG was positively related to C10 (D), C11 (**E**), and C18 (**F**), while negatively related to C28 (**G**), C29 (**H**) and C30 (**I**). C10 and C11: CD4 + T cell; C16 and C20: gamma-delta T cell; C18: double-negative T cell; C28 and C29: NK cell; C30: undefined cell group

To probe the potential relationships between antibody levels and the proportions of PBMC subpopulations, we generated scatter plots that illustrate the pairwise correlations between antibodies and subpopulations, and those with *r* > 0.5 and *p* < 0.05 are shown in Fig. 7. Notable observations include a positive correlation between C18 (DNT cells) and the anti-S IgM level, whereas C16 and C20 (gdT cells) display negative correlations. Additionally, C10 (CD4 + T cells), C11 (CD4 + T cells), and C18 (DNT cells) exhibit positive correlations with the anti-S IgG level. while C28 (NK cells), C29 (NK cells), and C30 (undefined) showed negative correlations, with the anti-S IgG level.

## Discussion

Numerous studies have substantiated the occurrence of lymphopenia subsequent to SARS-CoV-2 infection. Guan et al. compiled data from 1099 COVID-19 cases spanning 552 hospitals in China, revealing that 83.2% of patients experienced lymphopenia, with 36.2% encountering thrombocytopenia and 33.7% exhibiting leukopenia [15]. This phenomenon of lymphopenia has also been documented among critically ill COVID-19 patients in the United States [16]. Likewise, Fan et al. reported a significant reduction in baseline lymphocyte levels among patients in an ICU in Singapore [17].

Regarding the subsets of lymphocytes that are impaired during COVID-19 infection, a meta-analysis showed that the populations of CD4 + T cells, CD8 + T cells, B cells, and NK cells were all decreased, and this was especially pronounced for T cells and NK cells [18]. The systemic inflammatory response triggered by COVID-19 can lead to the release of copious cytokines, prompting a “cytokine storm” that may promote the apoptosis, pyroptosis, and necroptosis of lymphocytes [19, 20]. Additionally, lymphocytes might become entrapped in the spleen due to cytokine activation or sequestered in the lungs during extensive bilateral pneumonia [21, 22].

Upon viral RNA clearance, the recovery and immune reconstitution phases ensue. Various omics studies have been carried out in patients during the recovery period of COVID-19, and the changes in immunity have attracted common attention. Several studies have delved into the characteristics of PBMCs in patients recovering from COVID-19. Wen et al. categorized recovering COVID-19 patients into early recovery stage (ERS) and late recovery stage (LRS) [6], utilizing single-cell RNA sequencing analysis to characterize PBMC transcriptomic changes. ERS and LRS were defined as periods with blood samples free of nucleic acids within < 7 days and > 14 days, respectively. Their findings unveiled higher proportions of CD14 + monocytes, CD4 + TCM cells, CD8 + T cells, and plasma cells in recovered patients compared to healthy controls. In comparison to the ERS group, the LRS group exhibited elevated levels of CD4 + TEM cells, naïve CD4 + T cells, and naïve CD8 + T cells. Another study conducted by You et al. leveraged single-cell RNA sequencing analysis to compare healthy controls with individuals convalescing from COVID-19, discharged from the hospital for a minimum of 1 month [23]. This study showcased abundant TBET-enriched CD16 + and IRF1-enriched CD14 + monocytes in recovered individuals, alongside an accelerated development from immature B cells to antibody-producing plasma cells. In those two studies, the most affected subpopulations included CD14 + monocytes, naïve T cells and B cells, and CD4 + T cells, consistent with our results. Multi-omics studies are also ongoing. In one prospective study, 40-parameter mass cytometry, targeted proteomics and functional assays were performed at 6 and 12 months after infection. It found that by 6 months after infection, peripheral blood CD4 + T cell, CD8 + T cell, and NK cell counts had returned to normal levels in most patients and remained stable from 6 to 12 months, and the activation of CD4 + T cell and CD8 + T cell persisted up to 12 months after infection in patients with mild and severe COVID-19 [24]. The level of CD4 + T cell and CD8 + T cell, and NK cell counts at six months are similar to our results, and we complemented the change of immune cells at 1 week and 3 months after infection. Viral infection encompasses various impacts on the host, spanning immune cell responses, immune cell interactions, and antibody responses. In recovered COVID-19 patients, exploring immune cell composition shifts aids in comprehending dynamic immune changes post-recovery from virus infection, thereby guiding the protection of exposed individuals against COVID-19 reinfection.

In order to fathom the persistence of immune memory against SARS-CoV-2 after recovery, we embarked on exploring PBMC dynamics in COVID-19 patients within 6 months after viral RNA clearance. Research has shown that SARS-CoV-2-specific T-cell immunity can endure for 6 months post-infection [25]. Christian et al. observed that antibodies remained detectable 6 months after COVID-19 infection, although their numbers diminished, the number of memory B cells remained the same [26]. Our study may provide an explanation for the immune-cell response and showed that the composition of immune cells in patients differed according to the stage of the recovery period within 6 months, which has not been reported previously.

T cells wield the adaptive immune response against a range of pathogens. When pathogens invade, T cells rapidly mobilize to the site of infection. During the primary immune response, naive T cells are activated and proliferate clonally as effector cells to clear pathogens. Subsequent to antigen clearance, a fraction of antigen-specific T cells transition to memory T cells. Memory T cells, encompassing central memory (TCM), effector memory (TEM), and terminally differentiated effector memory (TEMRA) subsets, circulate within blood and tissues [27]. Understanding the circulation of memory T cells in blood and tissues can help us monitor the protective T cell responses in recovered COVID-19 patients. Our study initially utilized single-cell mass cytometry analysis to quantify shifts in T cell proportions and subsequently validated these findings using flow cytometry. Interestingly, we observed a decline in CD8 + TEMRA cells at the 6-month mark compared to 3 months after viral RNA clearance, while the remaining CD4 + and CD8 + T cell subsets were significantly different at 3 and 6 months of recovery and showed an upward trend. These findings are consistent with the observations made by Wiech et al., who identified an increase in CD8 + TEM cells at the 6-month juncture compared to 3 months post-viral RNA clearance [28]. Memory CD4 + and CD8 + T cells are endowed with the capability to guard against virus reinfection. Our results imply that memory T cells might offer protection against subsequent SARS-CoV-2 invasions, persisting for an extended period following recovery from COVID-19. Furthermore, this protective effect seems to be enhanced from 3 to 6 months post-recovery, potentially aiding in the clearance of SARS-CoV-2 from reservoir cells [29]. Terminal effector memory T cell is a specific subset that possesses limited proliferation capabilities but exerts potent effector functions. TEMRA cells display heightened cytotoxic gene expression, along with formidable killing and cytokine-releasing abilities [30]. Our findings revealed a slight decline in CD8 + TEMRA cell proportions at the 6-month mark subsequent to COVID-19 RNA clearance compared to 3 month. This phenomenon might be associated with TEMRA’s poor proliferation potential. As patients recuperate from COVID-19, cytokine levels including TNF-α, TGF-b, IFN-g, IL-1b, IL-2, and IL-4 subside [28]. Given that TEMRA differentiation hinges more on cytokines than antigenic stimulation, this shift could potentially contribute to the observed decrease in TEMRA proportions at the 6-month juncture post-viral RNA clearance, which further contributed to the decreased levels of cytokines in the recovery patients. The trajectory of cell phenotypes following COVID-19 infection was investigated in a study by Adamo et al., using spectral flow cytometry combined with cellular indexing of transcriptomes and T cell receptor sequencing, it was observed that there was a gradual transition from the T effector/TEM cell phenotype to a terminally differentiated TEMRA phenotype from 6 months to 1 year after infection. And it revealed that CD8 + TEMRA cells might constitute the main circulating memory subset after acute viral infection in humans [31]. Our study added to the picture of CD8 + TEMRA cells from 1 week to 6 months after viral RNA clearance, though the proportion of CD8 + TEMRA at 6 months after covid-19 RNA clearance was lower than that at 3 months, it was significantly higher than that at 1 week. As TEMRA cells wield effector functions designed to target latent viruses, the increased presence of CD8 + TEMRA cells at the 6 month may indicate their potential role in hindering the reactivation of latent COVID-19 in vivo over an extended period [32].

Notably, gdT cell proportions significantly increased, while DNT cell proportions declined during the recovery phase of COVID-19 patients, a phenomenon not hitherto documented. It is suggested that gdT cells could potentially serve a protective function in COVID-19 [33], gdT cells that were activated by SARS-CoV-2 can trigger a range of antiviral responses, including the release of cytokines, restriction of viral replication and cytolysis of virus-infected cells [34]. Massow et al. reported that COVID-19 patients with the most severe disease had the lowest numbers of gdT cells, but it was unclear how gdT cells affect prognosis [35]. Our results showed that compared with healthy people, a subset of gdT cells (C16) decreased significantly at T1 then increased at T2 and T3, while it was not present in C17 and C20, and there was a lower expression levels of CD57 in cluster 16 than other clusters. CD57 + CD4 + T cells and CD57 + CD8 + T cells are known to lack proliferative capacity, but gdT cells are different from αβT cells, CD57 is the surface receptor that distinguishes two cell types, Vδ2 T cells are CD57- [36]. Given that Vδ2 T cells represent the predominant subset of gdT cells in the peripheral blood, the observed alteration in C16 levels following COVID-19 infection in our study can be attributed to the utilization of PBMC samples. Lim et al. also found a similar phenomenon that the levels of Vδ2 TCM and Vδ2 TEM exhibited a sustained decline following COVID-19 infection, remaining below baseline levels during the late convalescence (39 days after infection) [37]. The changes in C16 indicate that it has a strong infiltration in the lungs in the early stages of COVID-19 recovery, which helps in clearing viruses in lungs and promoting the facilitating the reduction of inflammation [38]. DNT cells, constituting 1–3% of peripheral T cells, demonstrate immunoregulatory potential by eliminating B cells during chronic graft-versus-host disease [39]. In addition, DNT cells with some CD4 + T cell functions were shown to be associated with a nonpathogenic outcome following HIV infection. The role of DNT cell shifts in COVID-19 and their implications for the disease warrant further investigation. In the context of B cells, De Biasi et al. reported a decrease in the numbers of total and naïve B cells, as well as reduced proportions and numbers of memory switched and unswitched B cells in COVID-19 patients [40]. Interestingly, our study revealed a unique pattern in the behavior of B cell subsets. Within the initial 7 days after viral RNA clearance, both naïve and memory B cell numbers were notably higher. However, by the 3-month mark, these numbers exhibited a decline. This phenomenon might be attributed to the activation of B cells during the virus clearance phase, prompting proliferation, and subsequently, a gradual return to baseline levels during the recovery period. This nuanced dynamic of B cell populations sheds light on their role in the immune response during and after COVID-19 recovery. Additionally, Maucourant et al. have reported on distinct NK cell immunotypes characterized by the expression of perforin, NKG2C, and Ksp37. These immunotypes have demonstrated a correlation with COVID-19 disease severity [41]. The results indicated that although NK cells decreased in number, they were activated and functioned against several viral infections. CD14 + CD16 − monocytes are a classical monocyte subset, which was reported to be decreased in severe COVID-19 cases [42–44]. However, as patients transition toward recovery, the number of these cells gradually rises, indicating rebuilding of the immune system and stronger innate immunity and pathogen clearance capacity.

It is established that antibody levels targeting the SARS-CoV-2 S antigen wane post-viral RNA clearance or vaccination [13, 14, 45]. Similarly, we observed a decrease in both anti-S IgM and IgG antibody levels within 6 months after viral RNA clearance. We found the antibody levels correlated with CD4 + T cells, gdT cells, DNT cells, and NK cells. After SARS-CoV-2 infection, SARS-CoV-2 spike-specific memory B cells can rely on CD4 + T cells to produce high-affinity antibodies [46], the positive correlation between CD4 + T cells and anti-S IgG levels is understandable in our study. NK cells can secrete various cytokines, such as IFN-γ, TNF-α, etc. Kaneko et al. found that excessive TNF-α inhibited the formation of germinal center reactions, suppressed the generation of long-lasting antibody responses originating from germinal centers, resulting in the low and transient antibody responses in COVID-19 patients [47]. This may be one of the reasons for the negative correlation between NK cells and IgG antibody levels. Notably, DNT cells exhibited positive correlations with both IgM and IgG levels, which had not been reported previously. We speculated that it might be related to the limited long-term persistence of antibodies in COVID-19 patients. DNT cells have the ability of promoting B cell apoptosis, inhibiting B cell proliferatxion and plasma cell formation [48]. We hypothesized that after SARS-CoV-2 infection, when B cells produced antibodies, DNT cells might also continuously promote the apoptosis of B cells. This resulted in the situation that antibodies could not exist for a long time and could not provide long-term immune protection. However, this assumption still requires subsequent large-sample, dynamic exploration.

In T cells, except for naïve CD4 + T cells, which showed a decrease at 6 months after recovery compared to 7 days, other CD4 + T cells and CD8 + T cells maintained higher levels at 6 months after COVID-19 recovery. Similarly, NK cells also maintained higher levels at 6 months compared to 7 days after recovery. This is consistent with the widely recognized notion that there is relatively strong immune protection against COVID-19 during the first 3–6 months after the initial infection.While higher levels of T cells and NK cells provide protection against reinfection with the SARS-CoV-2 virus, it’s worth noting that currently, SARS-CoV-2 subvariants BQ.1 (a subvariant of BA.5) and XBB (a subvariant of BA.2) have globally replaced the previously dominant Omicron variant strains (including BA.5) [49]. Viral mutations resulting in immune escape increase the probability of reinfection. After infection, the increase in the number of naïve and memory B cells is sustained for a relatively short period before declining, followed by a gradual return to levels close to those in uninfected individuals by 6 months after viral RNA clearance, suggests that their protective role against reinfection in the event of a new COVID-19 exposure may be relatively limited.

This is the first study to examine the changes in PBMCs in patients with COVID-19 within 6 months after viral RNA clearance. Although, the small number of patients enrolled in this study partially limits the reliability of the experimental results, longitudinal dynamic observations revealed consistent changes across different samples. Another limitation of this study is that the biological functions of the most altered subsets of PBMCs were not investigated in detail, and further studies are required to determine the significance of these changes to the host immune status.Furthermore, selecting 6 months as the observation endpoint, longer-term changes in immune cell profiles after clearance of COVID-19 RNA are also worth investigating.

In conclusion, we described longitudinal dynamic changes in the PBMCs of COVID-19 patients within 6 months after viral RNA clearance. Our results identified distinct changes in the subpopulations of T cells, B cells, NK cells, and monocytes. In addition, we described possible relationships between anti-S antibodies and several subsets of immune cells, which could help elucidate the immune changes that occur during COVID-19 recovery. Our study provides a strategy for guiding prevention against reinfection in the current scenario of continuously changing COVID-19 virus strains.

## Electronic supplementary material

Below is the link to the electronic supplementary material.

Supplementary Material 1

## Data Availability

The original contributions presented in the study are included in the article/supplementary material, further inquiries can be directed to the corresponding author/s.
