# Correction: Longitudinal dynamic single-cell mass cytometry analysis of peripheral blood mononuclear cells in COVID-19 patients within 6 months after viral RNA clearance

**DOI:** 10.1186/s12879-024-09543-2

**Published:** 2024-09-26

**Authors:** Diwenxin Zhou, Shuai Zhao, Keting He, Qiuhong Liu, Fen Zhang, Zhangya Pu, Lanlan Xiao, Lingjian Zhang, Shangci Chen, Xiaohan Qian, Xiaoxin Wu, Yangfan Shen, Ling Yu, Huafen Zhang, Jiandi Jin, Min Xu, Xiaoyan Wang, Danhua Zhu, Zhongyang Xie, Xiaowei Xu

**Affiliations:** State Key Laboratory for Diagnosis and Treatment of Infectious Diseases, National Clinical Research Center for Infectious Diseases, National Medical Center for Infectious Diseases, Collaborative Innovation Center for Diagnosis and Treatment of Infectious Diseases, The First Affiliated Hospital, Zhejiang University School of Medicine, 79 Qingchun Rd., Hangzhou City, 310003 China

Following publication of the original article [1], we have been notified that the first affiliation needs to be corrected to:

State Key Laboratory for Diagnosis and Treatment of Infectious Diseases, National Clinical Research Center for Infectious Diseases, National Medical Center for Infectious Diseases, Collaborative Innovation Center for Diagnosis and Treatment of Infectious Diseases, The First Affiliated Hospital, Zhejiang University School of Medicine,79 Qingchun Rd., Hangzhou City 310,003, China.

Originally published affiliation: State Key Laboratory for Diagnosis and Treatment of Infectious Diseases, National Clinical Research Center for Infectious Diseases, Collaborative Innovation Center for Diagnosis and Treatment of Infectious Diseases, College of Medicine, The First Affiliated Hospital, Zhejiang University, Hangzhou, China.

The original article has been corrected.
