# Supplementary material for: Longitudinal dynamic single-cell mass cytometry analysis of peripheral blood mononuclear cells in COVID-19 patients within 6 months after viral RNA clearance
Source: BMC Infect Dis. 2024 Jun 6;24:567. doi: 10.1186/s12879-024-09464-0 (PMC11157885; doi:10.1186/s12879-024-09464-0)
Supplement: Supplementary file 1 — Supplementary Material 1 [file 12879_2024_9464_MOESM1_ESM.docx]

**Supplementary methods**

## CyTOF

### Single-cell suspension preparation

PBMCs were resuspended in 5 mL ice-cold cell staining buffer. The cells were collected by centrifugation at 300 × *g* for 5 min at 2–8°C. After removing the supernatant, the cells were resuspended in cell staining buffer and counted.

### Antibodies

For mass cytometric analysis, purified antibodies were obtained from BioLegend (San Diego, CA, USA), eBioscience (San Diego, CA, USA), Bio X Cell (Lebanon, NH, USA), R&D Systems (Minneapolis, MN, USA), and BD Biosciences (Franklin Lakes, NJ, USA) using the clones listed in **Supplementary Table 1**. A total of 31 cell-surface antibodies were used to identify the subpopulations of PBMCs. Antibody labeling with the indicated metal tag was performed using the Maxpar Antibody Labeling Kit (Fluidigm, South San Francisco, CA, USA). Conjugated antibodies were titrated to the optimal concentration before use.

### Mass cytometry, staining, and data acquisition

Cells were washed once with 1× PBS, stained with 100 μL 250 nM cisplatin (Fluidigm) for 5 min on ice to exclude dead cells, and then incubated in Fc receptor blocking solution before staining with a surface antibody cocktail for 30 min on ice. Cells were washed twice with FACS buffer (1× PBS + 0.5% BSA) and fixed in 200 μL intercalation solution (Maxpar Fix and Perm Buffer containing 250 nM ^191/193^Ir; Fluidigm) overnight. After fixation, cells were washed once with FACS buffer and then permeabilization buffer (eBioscience) and stained with intracellular antibody cocktail for 30 min on ice. Cells were washed and resuspended in deionized water, followed by addition of 20% EQ beads (Fluidigm), and then evaluated by mass cytometry (Helios; Fluidigm).

### CyTOF data analysis

Raw data from each sample were de-barcoded using a doublet-filtering scheme with unique mass-tagged barcodes. Each .fcs file generated from different batches was normalized using a bead normalization method. Data were manually gated using FlowJo software (FlowJo, Ashland, OR, USA) to exclude debris, dead cells, and doublets, leaving single live immune cells. The X-shift clustering algorithm was applied to partition the cells into distinct phenotypes based on marker expression levels. The cell type of each cluster was annotated according to the marker expression pattern on a cluster versus marker heatmap. The t-distributed stochastic neighbor embedding (t-SNE) dimensionality reduction algorithm was used to visualize the high-dimensional data in two dimensions and to show the distribution of each cluster and marker expression and differences among the groups or different sample types. The frequencies of the annotated cell populations were analyzed using the *t* test.

Supplementary Table 1 Markers information used for cyTOF analysis.

| List | Marker | Metal | Clone | Manufacturer | List | Marker | Metal | Clone | Manufacturer |
| --- | --- | --- | --- | --- | --- | --- | --- | --- | --- |
| 1 | CD45 | 89Y | HI30 | Biolegend | 17 | CTLA4 | 161Dy | 14D3 | Bioxcell |
| 2 | CD3 | 115In | UCHT1 | Bioxcell | 18 | CD161 | 141Eu | HP-3G10 | Biolegend |
| 3 | CD56 | 141Pr | NCAM16.2 | BD | 19 | CXCR5 | 155Gd | RF8B2 | BD |
| 4 | CCR6 | 143Nd | G034E3 | Biolegend | 20 | TCR_gd | 142Nd | 5A6.E9 | PLT |
| 5 | CD14 | 144Nd | M5E2 | Biolegend | 21 | CXCR3 | 157Gd | G025H7 | Biolegend |
| 6 | IgD | 171Yb | IA6-2 | Biolegend | 22 | CD57 | 153Eu | HNK-1 | Biolegend |
| 7 | CD19 | 142Nd | HIB19 | Biolegend | 23 | CD127 | 170Er | A019D5 | Biolegend |
| 8 | CD25 | 149Sm | 24212 | BD | 24 | PD1 | 174Yb | EH12.2H7 | Biolegend |
| 9 | ICOS | 150Nd | C398.4A | Biolegend | 25 | CD38 | 146Nd | HIT2 | Biolegend |
| 10 | CD27 | 145Nd | O323 | Biolegend | 26 | CCR4 | 169Tm | L291H4 | Biolegend |
| 11 | CD45RA | 167Er | HI100 | Biolegend | 27 | CD16 | 175Lu | 3G8 | Biolegend |
| 12 | CD86 | 156Gd | Fun-1 | BD | 28 | HLA_DR | 176Yb | L243 | Biolegend |
| 13 | CD28 | 148Nd | CD28.2 | Biolegend | 29 | CD4 | 197Au | RPA-T4 | Biolegend |
| 14 | CCR7 | 147Sm | G043H7 | Biolegend | 30 | CD8 | 198Pt | RPA-T8 | Biolegend |
| 15 | CD11c | 159Tb | BU15 | Biolegend | 31 | CD11b | 209Bi | M1/70 | Biolegend |
| 16 | CD33 | 160Gd | WM53 | Biolegend |  |  |  |  |  |

Supplementary Table 2 Markers information used for polychromatic flow cytometry.

| List | Marker | Fluorochrome | Clone | Manufacturer |
| --- | --- | --- | --- | --- |
| 1 | CD45RA | FITC | HI100 | BD |
| 2 | CD3 | APC-H7 | SK7 | BD |
| 3 | CD4 | BV510 | SK3 | BD |
| 4 | CD8 | APC | RPA-T8 | BD |
| 5 | CCR7 | BV421 | 150503 | BD |

Supplementary Table 3 Characteristics of enrolled healty controls and patients at admission analysed by CyTOF

|  | Control 1 | Control 2 | Control 3 | Control 4 | Patient 1 | Patient 2 | Patient 3 | Patient 4 | Patient 5 | Patient 6 |
| --- | --- | --- | --- | --- | --- | --- | --- | --- | --- | --- |
| Age | 49 | 47 | 56 | 50 | 64 | 51 | 48 | 59 | 52 | 45 |
| Sex (Male or Female) | F | M | F | M | F | M | M | F | M | F |
| Symptoms（yes or no） |  |  |  |  |  |  |  |  |  |  |
| Fever |  |  |  |  | Y | Y | Y | Y | Y | Y |
| Cough |  |  |  |  | Y | Y | N | Y | Y | Y |
| Fatigue |  |  |  |  | N | Y | Y | N | N | Y |
| Muscle ache |  |  |  |  | Y | N | Y | N | Y | N |
| Dyspnea |  |  |  |  | N | Y | N | N | N | N |
| Diarrhea |  |  |  |  | N | N | N | N | N | N |
| Blood test |  |  |  |  |  |  |  |  |  |  |
| White blood counts (*10^9/L) |  |  |  |  | 2.10 | 4.00 | 17.6 | 6 | 3.50 | 5.89 |
| Leukocytes (*10^9/L) |  |  |  |  | 1.80 | 2.52 | 16.5 | 3.8 | 2.10 | 4.56 |
| Lymphocytes (*10^9/L) |  |  |  |  | 0.20 | 1.07 | 0.63 | 1.8 | 1.10 | 0.92 |
| Hemoglobin (g/L) |  |  |  |  | 142 | 162 | 133 | 122 | 135 | 144 |
| Plateles (*10^9/L) |  |  |  |  | 80 | 121 | 367 | 239 | 145 | 164 |
| INR |  |  |  |  | 0.95 | 1.17 | 0.93 | 0.99 | 0.93 | 1.15 |
| ALT |  |  |  |  | 10 | 20 | 287 | 51 | 17 | 19 |
| AST |  |  |  |  | 20 | 30 | 111 | 39 | 38 | 28.3 |
| Albumin |  |  |  |  | 43.60 | 36.20 | 35.9 | 45.3 | 35.10 | 35.20 |
| LDH |  |  |  |  | 246 | 312 | 361 | 191 | 310 | 187 |
| Creatinine |  |  |  |  | 66 | 81 | 75 | 62 | 77 | 57 |
| C-creative protein |  |  |  |  | 3.55 | 42.45 | 89 | 0.87 | 7.50 | 107.10 |
| Bilateral pneumonia on CT scan |  |  |  |  | Y | Y | Y | Y | N | Y |
| Severity |  |  |  |  | Mild | Severe | Severe | Mild | Mild | Severe |
| Time from onset to RNA shedding (days) |  |  |  |  | 14 | 22 | 16 | 13 | 15 | 24 |

Abbreviation: ALT, Alanine transaminase; AST, Aspartate Aminotransferase; BMI, Body Mass Index; INR, International Normalized Ratio; LDH, lactate dehydrogenase.

Supplemetary Table 4 Characteristics of enrolled patients at admission analysed by flow cytometry

|  | Patient 1 | Patient 2 | Patient 3 | Patient 4 | Patient 5 | Patient 6 | Patient 7 | Patient 8 | Patient 9 | Patient 10 |
| --- | --- | --- | --- | --- | --- | --- | --- | --- | --- | --- |
| Group | T2 | T2 | T2 | T2 | T2 | T2 | T2 | T2 | T2 | T2 |
| Age | M | M | M | M | M | F | F | M | M | F |
| Sex (Male or Female) | 63 | 71 | 36 | 57 | 73 | 58 | 44 | 30 | 60 | 49 |
| Symptoms（yes or no） |  |  |  |  |  |  |  |  |  |  |
| Fever | N | Y | Y | Y | Y | Y | Y | Y | Y | N |
| Cough | Y | Y | N | N | Y | Y | Y | N | Y | Y |
| Fatigue | N | N | N | N | N | N | N | N | N | N |
| Muscle ache | N | N | Y | N | N | N | N | N | N | N |
| Dyspnea | N | Y | N | N | N | N | N | N | Y | N |
| Diarrhea | N | N | N | N | N | N | N | N | N | N |
| Blood test |  |  |  |  |  |  |  |  |  |  |
| White blood counts (*10^9/L) | 6.5 | 10.8 | 5.1 | 7.5 | 2.8 | 8 | 4.2 | 4.2 | 7.3 | 4 |
| Leukocytes (*10^9/L) | 91.3 | 90.9 | 68.8 | 88.8 | 90.1 | 85.4 | 68.8 | 52.9 | 88.1 | 61.6 |
| Lymphocytes (*10^9/L) | 6.6 | 3.5 | 23.1 | 7.6 | 7.8 | 7.5 | 19.9 | 32.5 | 8.4 | 25 |
| Hemoglobin (g/L) | 132 | 141 | 144.00 | 146.00 | 112 | 119.00 | 140 | 156 | 143.00 | 141 |
| Plateles (*10^9/L) | 243 | 195 | 187.00 | 300.00 | 189 | 170.00 | 226 | 166 | 165.00 | 208 |
| INR | 1.04 | 0.94 | 1.09 | 0.87 | 1.1 | 1.11 | 1.05 | 0.95 | 1.05 | 0.92 |
| ALT | 9 | 23 | 25.00 | 15 | 32 | 27.00 | 12 | 22 | 40.00 | 52 |
| AST | 19 | 18 | 23.00 | 22 | 44 | 32.00 | 16 | 20 | 40.00 | 22 |
| Albumin | 38.3 | 29.7 | 43.6 | 36.1 | 37 | 37 | 49.5 | 45.9 | 38.2 | 44.4 |
| LDH | 228 | 267 | 276 | 286 | 448 | 237 | 215 | 213 | 233 | 177 |
| Creatinine | 77 | 83 | 89 | 68 | 66 | 53 | 78 | 103 | 72 | 64 |
| C-creative protein | 7.47 | 9.16 | 10.33 | 3.83 | 107.04 | 2.17 | 5.31 | 15.11 | 0.8 | 9.89 |
| Bilateral pneumonia on CT scan | Y | N | Y | N | N | Y | Y | Y | N | Y |
| Severity | Severe | Severe | Severe | Severe | Severe | Mild | Mild | Mild | Severe | Mild |
| Time from onset to RNA shedding (days) | 44 | 23 | 13 | 13 | 18 | 38 | 7 | 14 | 16 | 38 |

|  | Patient 11 | Patient 12 | Patient 13 | Patient 14 | Patient 15 | Patient 16 | Patient 17 | Patient 18 | Patient 19 | Patient 20 |
| --- | --- | --- | --- | --- | --- | --- | --- | --- | --- | --- |
| Group | T2 | T2 | T2 | T2 | T2 | T2 | T2 | T2 | T2 | T2 |
| Age | M | F | M | M | M | F | F | M | F | M |
| Sex (Male or Female) | 61 | 46 | 71 | 54 | 30 | 59 | 39 | 49 | 31 | 67 |
| Symptoms（yes or no） |  |  |  |  |  |  |  |  |  |  |
| Fever | N | Y | Y | Y | Y | Y | Y | Y | N | Y |
| Cough | N | Y | N | N | Y | Y | Y | Y | Y | N |
| Fatigue | Y | N | Y | N | N | N | N | N | N | N |
| Muscle ache | N | N | Y | Y | N | N | N | N | N | Y |
| Dyspnea | N | N | N | N | Y | Y | N | N | N | N |
| Diarrhea | Y | N | N | N | N | N | N | N | N | N |
| Blood test |  |  |  |  |  |  |  |  |  |  |
| White blood counts (*10^9/L) | 13 | 1.6 | 13 | 3.6 | 21.2 | 4.1 | 13.3 | 19 | 8.7 | 3.7 |
| Leukocytes (*10^9/L) | 91.9 | 74.9 | 92.3 | 61.2 | 89.4 | 66.7 | 82.5 | 93.7 | 81.4 | 74.5 |
| Lymphocytes (*10^9/L) | 4.6 | 19.6 | 3.9 | 27.2 | 4 | 22.4 | 11.3 | 4.8 | 12.9 | 14.9 |
| Hemoglobin (g/L) | 145 | 137.00 | 128 | 154 | 148 | 137 | 129 | 148.00 | 114 | 155.00 |
| Plateles (*10^9/L) | 131 | 146.00 | 174 | 137 | 195 | 165 | 173 | 171.00 | 96 | 176.00 |
| INR | 0.94 | 1.00 | 0.93 | 0.99 | 0.99 | 1.06 | 0.89 | 1.01 | 0.9 | 1.08 |
| ALT | 10 | 11.00 | 26 | 18 | 23 | 13 | 22 | 47.00 | 15 | 26.00 |
| AST | 14 | 18.00 | 26 | 22 | 15 | 16 | 14 | 38.00 | 19 | 22.00 |
| Albumin | 29.7 | 39.1 | 27.1 | 40.4 | 36.1 | 36.1 | 38.9 | 28.4 | 32.4 | 44.2 |
| LDH | 363 | 220 | 275 | 197 | 211 | 275 | 202 | 235 | 208 | 223 |
| Creatinine | 118 | 49 | 61 | 79 | 91 | 59 | 68 | 69 | 55 | 95 |
| C-creative protein | 4.98 | 7.95 | 69.35 | 87.34 | 4 | 20.2 | 0.48 | 39.48 | 11.76 | 51.61 |
| Bilateral pneumonia on CT scan | Y | N | Y | N | Y | Y | Y | Y | Y | Y |
| Severity | Severe | Mild | Severe | Severe | Severe | Mild | Mild | Mild | Mild | Severe |
| Time from onset to RNA shedding (days) | 7 | 18 | 7 | 28 | 5 | 12 | 22 | 15 | 5 | 13 |

|  | Patient 1 | Patient 2 | Patient 3 | Patient 4 | Patient 5 | Patient 6 | Patient 7 | Patient 8 | Patient 9 | Patient 10 |
| --- | --- | --- | --- | --- | --- | --- | --- | --- | --- | --- |
| Group | T3 | T3 | T3 | T3 | T3 | T3 | T3 | T3 | T3 | T3 |
| Age | F | M | M | F | M | F | M | M | M | M |
| Sex (Male or Female) | 25 | 30 | 60 | 44 | 36 | 67 | 71 | - | 30 | 54 |
| Symptoms（yes or no） |  |  |  |  |  |  |  |  |  |  |
| Fever | Y | Y | Y | Y | Y | Y | Y | - | Y | Y |
| Cough | N | N | Y | Y | N | Y | Y | - | Y | N |
| Fatigue | N | N | N | N | N | N | N | - | N | N |
| Muscle ache | N | N | N | N | Y | N | N | - | N | Y |
| Dyspnea | N | N | Y | N | N | N | Y | - | Y | N |
| Diarrhea | N | N | N | N | N | N | N | - | N | N |
| Blood test |  |  |  |  |  |  |  |  |  |  |
| White blood counts (*10^9/L) | 6.6 | 4.2 | 7.3 | 4.2 | 5.1 | 7.9 | 10.8 | - | 21.2 | 3.6 |
| Leukocytes (*10^9/L) | 56.4 | 52.9 | 88.1 | 68.8 | 68.8 | 92.2 | 90.9 | - | 89.4 | 61.2 |
| Lymphocytes (*10^9/L) | 22.7 | 32.5 | 8.4 | 19.9 | 23.1 | 6.2 | 3.5 | - | 4 | 27.2 |
| Hemoglobin (g/L) | 132 | 156 | 143.00 | 140 | 144.00 | 109 | 141 | - | 148 | 154 |
| Plateles (*10^9/L) | 318 | 166 | 165.00 | 226 | 187.00 | 402 | 195 | - | 195 | 137 |
| INR | 1.1 | 0.95 | 1.05 | 1.05 | 1.09 | 0.93 | 0.94 | - | 0.99 | 0.99 |
| ALT | 100 | 22 | 40.00 | 12 | 25.00 | 27 | 23 | - | 23 | 18 |
| AST | 41 | 20 | 40.00 | 16 | 23.00 | 25 | 18 | - | 15 | 22 |
| Albumin | 40.5 | 45.9 | 38.2 | 49.5 | 43.6 | 31.4 | 29.7 | - | 36.1 | 40.4 |
| LDH | 182 | 213 | 233 | 215 | 276 | 373 | 267 | - | 211 | 197 |
| Creatinine | 54 | 103 | 72 | 78 | 89 | 44 | 83 | - | 91 | 79 |
| C-creative protein | 3.54 | 15.11 | 0.8 | 5.31 | 10.33 | 136.95 | 9.16 | - | 4 | 87.34 |
| Bilateral pneumonia on CT scan | Y | Y | N | Y | Y | N | N | - | Y | N |
| Severity | Mild | Mild | Severe | Mild | Severe | Severe | Severe | - | Severe | Severe |
| Time from onset to RNA shedding (days) | 14 | 14 | 16 | 7 | 13 | 14 | 23 | - | 5 | 28 |

|  | Patient 11 | Patient 12 | Patient 13 | Patient 14 | Patient 15 | Patient 16 | Patient 17 |
| --- | --- | --- | --- | --- | --- | --- | --- |
| Group | T3 | T3 | T3 | T3 | T3 | T3 | T3 |
| Age | F | F | F | M | M | M | M |
| Sex (Male or Female) | 46 | 55 | 39 | 31 | 47 | 56 | 49 |
| Symptoms（yes or no） |  |  |  |  |  |  |  |
| Fever | Y | Y | Y | Y | Y | Y | Y |
| Cough | Y | N | Y | Y | Y | Y | Y |
| Fatigue | N | Y | N | N | N | N | Y |
| Muscle ache | N | N | N | N | N | N | N |
| Dyspnea | N | N | N | N | N | N | N |
| Diarrhea | N | N | N | N | N | N | N |
| Blood test |  |  |  |  |  |  |  |
| White blood counts (*10^9/L) | 1.6 | 11.8 | 13.3 | 5.9 | 2.8 | 16.3 | 5.7 |
| Leukocytes (*10^9/L) | 74.9 | 89.4 | 82.5 | 73.4 | 61.1 | 91.6 | 87.5 |
| Lymphocytes (*10^9/L) | 19.6 | 7.6 | 11.3 | 12.6 | 31 | 5.1 | 10.4 |
| Hemoglobin (g/L) | 137.00 | 138 | 129 | 167 | 163 | 150 | 150 |
| Plateles (*10^9/L) | 146.00 | 112 | 173 | 187 | 143 | 162 | 191 |
| INR | 1.00 | 1 | 0.89 | 1.03 | 1 | 0.9 | 0.96 |
| ALT | 11.00 | 17 | 22 | 57 | 35 | 16 | 39 |
| AST | 18.00 | 29 | 14 | 34 | 23 | 18 | 42 |
| Albumin | 39.1 | 35.4 | 38.9 | 46.6 | 34.5 | 37.5 | 38.2 |
| LDH | 220 | 306 | 202 | 191 | 484 | 163 | 421 |
| Creatinine | 49 | 63 | 68 | 108 | 74 | 75 | 61 |
| C-creative protein | 7.95 | 11.39 | 0.48 | 1.7 | 35.26 | 13.33 | 54.01 |
| Bilateral pneumonia on CT scan | N | Y | Y | Y | Y | Y | Y |
| Severity | Mild | Severe | Mild | Mild | Severe | Mild | Severe |
| Time from onset to RNA shedding (days) | 18 | 10 | 22 | 23 | 10 | 18 | 5 |

Abbreviation: ALT, Alanine transaminase; AST, Aspartate Aminotransferase; BMI, Body Mass Index; INR, International Normalized Ratio; LDH, lactate dehydrogenase.

Supplemetary Table 5 Proportion of different cell groups in each sample analysed by CyTOF

| Sample | group | Frequancy of different immune cell subpopulation | | | | | | | | | |
| --- | --- | --- | --- | --- | --- | --- | --- | --- | --- | --- | --- |
|  |  | CD8+T | DNT | CD4+T | NKT | gdT | undefined | B cell | NK | Monocytes | DC |
| H1 | HC | 13.42533 | 0.332689 | 24.66931 | 0.004573 | 1.191279 | 1.862374 | 21.25553 | 22.62402 | 13.60253 | 1.032366 |
| H2 | HC | 21.78159 | 0.157222 | 21.02616 | 0.371324 | 15.10862 | 2.042603 | 9.853835 | 14.16273 | 14.85553 | 0.640391 |
| H3 | HC | 7.191898 | 1.696736 | 35.44202 | 0.02225 | 35.02816 | 1.666222 | 4.94145 | 4.407446 | 8.978907 | 0.624913 |
| H4 | HC | 14.81985 | 0.281235 | 19.12532 | 0.063855 | 1.703712 | 3.094261 | 11.26569 | 33.6911 | 14.19148 | 1.763491 |
| P1 | T1 | 16.24784 | 2.106583 | 37.60577 | 0.625466 | 0.098424 | 2.278031 | 13.72533 | 5.719683 | 20.75627 | 0.836601 |
| P2 | T1 | 20.35992 | 0.986025 | 19.10925 | 0.169887 | 0.050368 | 6.932908 | 11.36276 | 12.27878 | 27.99969 | 0.750403 |
| P3 | T1 | 9.092046 | 1.063204 | 16.13936 | 0.027224 | 0.059598 | 4.213082 | 12.52814 | 30.72695 | 25.39916 | 0.751232 |
| P4 | T1 | 12.3495 | 5.984295 | 30.16462 | 0.1722 | 0.280342 | 2.30197 | 5.523488 | 3.063783 | 38.62309 | 1.536713 |
| P5 | T1 | 6.254088 | 0.791016 | 9.052878 | 0.017276 | 0.013574 | 2.460665 | 16.03875 | 2.389091 | 61.85599 | 1.126674 |
| P6 | T1 | 7.45234 | 0.990344 | 5.706858 | 0.074276 | 0.099034 | 33.36222 | 8.875959 | 4.753652 | 34.21639 | 4.468928 |
| P1 | T2 | 25.45984 | 0.995364 | 17.10341 | 1.48444 | 8.690594 | 0.992397 | 5.887311 | 19.26864 | 18.84664 | 1.27136 |
| P2 | T2 | 17.04922 | 0.255937 | 18.14079 | 0.17997 | 3.658184 | 2.108995 | 3.607539 | 24.20008 | 29.7267 | 1.072585 |
| P3 | T2 | 23.13987 | 0.228282 | 19.96462 | 0.15953 | 1.726796 | 0.909789 | 4.609685 | 32.5922 | 15.52849 | 1.14074 |
| P4 | T2 | 8.433906 | 0.101884 | 11.05592 | 0.00899 | 0.670487 | 4.985579 | 8.505075 | 39.57224 | 25.93625 | 0.72967 |
| P5 | T2 | 16.00666 | 3.096359 | 38.4558 | 0.16651 | 12.47955 | 0.810106 | 5.531745 | 7.217114 | 15.3884 | 0.847752 |
| P6 | T2 | 11.31143 | 0.138588 | 7.694938 | 0.030597 | 2.20541 | 3.340513 | 9.268003 | 42.04798 | 22.67145 | 1.291089 |
| P1 | T3 | 23.57939 | 0.264172 | 20.58771 | 1.881885 | 7.109495 | 2.94402 | 6.912047 | 14.24895 | 20.7838 | 1.688522 |
| P2 | T3 | 21.43373 | 0.268561 | 18.67842 | 0.329307 | 3.707422 | 4.745858 | 5.500387 | 27.38939 | 17.25761 | 0.689307 |
| P3 | T3 | 20.64029 | 0.234425 | 24.34958 | 0.148831 | 1.388667 | 2.134102 | 6.376754 | 27.37156 | 16.17982 | 1.17596 |
| P4 | T3 | 11.03775 | 0.262198 | 23.62406 | 0.012713 | 1.551736 | 3.468961 | 8.03121 | 33.39849 | 17.95104 | 0.661852 |
| P5 | T3 | 15.66708 | 2.217815 | 40.57588 | 0.145918 | 9.358597 | 2.145564 | 8.908093 | 5.917478 | 14.20081 | 0.862759 |
| P6 | T3 | 15.59619 | 0.614297 | 23.39996 | 0.031175 | 3.56533 | 2.061826 | 9.742307 | 24.23177 | 19.81337 | 0.943764 |

Supplementary Table 6 Proportion of different cell groups in each sample analysed by flow cytometry

| Sample | group | Frequancy of different T cell subpopulation | | | | | | | |
| --- | --- | --- | --- | --- | --- | --- | --- | --- | --- |
|  |  | CD4+T | CD4+ naive T cell | CD4+TCM | CD4+TEM | CD8+T | CD8+ naive T cell | CD8+TEM | CD8+TEMRA |
| S1 | T2 | 0.1830 | 0.0089 | 0.0810 | 0.0709 | 0.2300 | 0.0177 | 0.0686 | 0.1350 |
| S2 | T2 | 0.2760 | 0.1510 | 0.1070 | 0.0134 | 0.1880 | 0.0021 | 0.0631 | 0.1230 |
| S3 | T2 | 0.1850 | 0.0868 | 0.0719 | 0.0233 | 0.2240 | 0.0441 | 0.0581 | 0.1140 |
| S4 | T2 | 0.1800 | 0.0521 | 0.0865 | 0.0355 | 0.2900 | 0.0134 | 0.0235 | 0.2460 |
| S5 | T2 | 0.2160 | 0.0929 | 0.0590 | 0.0613 | 0.3960 | 0.0022 | 0.0435 | 0.3490 |
| S6 | T2 | 0.2190 | 0.1610 | 0.0417 | 0.0136 | 0.1880 | 0.0711 | 0.0174 | 0.0952 |
| S7 | T2 | 0.1160 | 0.0562 | 0.0351 | 0.0239 | 0.3620 | 0.0123 | 0.0699 | 0.2720 |
| S8 | T2 | 0.1580 | 0.0802 | 0.0647 | 0.0107 | 0.2310 | 0.0644 | 0.0626 | 0.0969 |
| S9 | T2 | 0.1060 | 0.0567 | 0.0402 | 0.0069 | 0.1450 | 0.0402 | 0.0277 | 0.0754 |
| S10 | T2 | 0.2480 | 0.0936 | 0.0909 | 0.0590 | 0.1890 | 0.0478 | 0.0123 | 0.1250 |
| S11 | T2 | 0.1140 | 0.0504 | 0.0419 | 0.0204 | 0.2490 | 0.0153 | 0.0921 | 0.1360 |
| S12 | T2 | 0.2600 | 0.1650 | 0.0663 | 0.0261 | 0.2790 | 0.0792 | 0.0476 | 0.1470 |
| S13 | T2 | 0.2380 | 0.1220 | 0.0102 | 0.0442 | 0.3050 | 0.0132 | 0.0470 | 0.2440 |
| S14 | T2 | 0.2010 | 0.0231 | 0.0853 | 0.0903 | 0.3810 | 0.0046 | 0.1520 | 0.2140 |
| S15 | T2 | 0.1880 | 0.0929 | 0.0689 | 0.0253 | 0.3820 | 0.0727 | 0.1290 | 0.1730 |
| S16 | T2 | 0.1890 | 0.1350 | 0.0478 | 0.0060 | 0.4100 | 0.0228 | 0.0140 | 0.3690 |
| S17 | T2 | 0.2140 | 0.1590 | 0.0346 | 0.0185 | 0.1660 | 0.0223 | 0.0362 | 0.1060 |
| S18 | T2 | 0.1330 | 0.0551 | 0.0552 | 0.0206 | 0.2460 | 0.0580 | 0.0742 | 0.1100 |
| S19 | T2 | 0.2850 | 0.1220 | 0.0806 | 0.0681 | 0.3320 | 0.0233 | 0.0698 | 0.2380 |
| S20 | T3 | 0.3140 | 0.2130 | 0.0940 | 0.0057 | 0.2550 | 0.0488 | 0.0123 | 0.1860 |
| S21 | T3 | 0.2910 | 0.2240 | 0.0482 | 0.0163 | 0.2050 | 0.0469 | 0.0387 | 0.1160 |
| S22 | T3 | 0.2450 | 0.1040 | 0.1120 | 0.0267 | 0.2670 | 0.1150 | 0.0721 | 0.0642 |
| S23 | T3 | 0.3290 | 0.1710 | 0.1320 | 0.0218 | 0.2170 | 0.0610 | 0.0626 | 0.0878 |
| S24 | T3 | 0.4970 | 0.2190 | 0.2280 | 0.0489 | 0.2980 | 0.0970 | 0.0800 | 0.0955 |
| S25 | T3 | 0.2550 | 0.0988 | 0.1230 | 0.0307 | 0.2600 | 0.0599 | 0.0742 | 0.1150 |
| S26 | T3 | 0.3860 | 0.1050 | 0.1600 | 0.1100 | 0.2340 | 0.0031 | 0.1340 | 0.0956 |
| S27 | T3 | 0.3550 | 0.1720 | 0.1150 | 0.0520 | 0.2160 | 0.0039 | 0.0819 | 0.1290 |
| S28 | T3 | 0.3930 | 0.2020 | 0.1280 | 0.0598 | 0.2540 | 0.0980 | 0.0599 | 0.0849 |
| S29 | T3 | 0.2520 | 0.0981 | 0.0957 | 0.0531 | 0.3590 | 0.0788 | 0.1250 | 0.1460 |
| S30 | T3 | 0.2810 | 0.0525 | 0.1300 | 0.0963 | 0.3530 | 0.0145 | 0.1310 | 0.1930 |
| S31 | T3 | 0.2670 | 0.1250 | 0.0958 | 0.0443 | 0.2290 | 0.0468 | 0.0568 | 0.1170 |
| S32 | T3 | 0.2240 | 0.1120 | 0.0805 | 0.0297 | 0.2520 | 0.0766 | 0.0650 | 0.1020 |
| S33 | T3 | 0.4720 | 0.2760 | 0.1640 | 0.0304 | 0.1950 | 0.0713 | 0.0415 | 0.0744 |
| S34 | T3 | 0.2810 | 0.0930 | 0.0993 | 0.0821 | 0.3910 | 0.1430 | 0.1100 | 0.1240 |
| S35 | T3 | 0.2720 | 0.1410 | 0.0902 | 0.0379 | 0.2100 | 0.0606 | 0.0652 | 0.0811 |
| S36 | T3 | 0.3900 | 0.1960 | 0.1190 | 0.0714 | 0.2820 | 0.0980 | 0.0647 | 0.1020 |
| S37 | T3 | 0.2330 | 0.0864 | 0.0774 | 0.0604 | 0.4010 | 0.1450 | 0.1250 | 0.1230 |


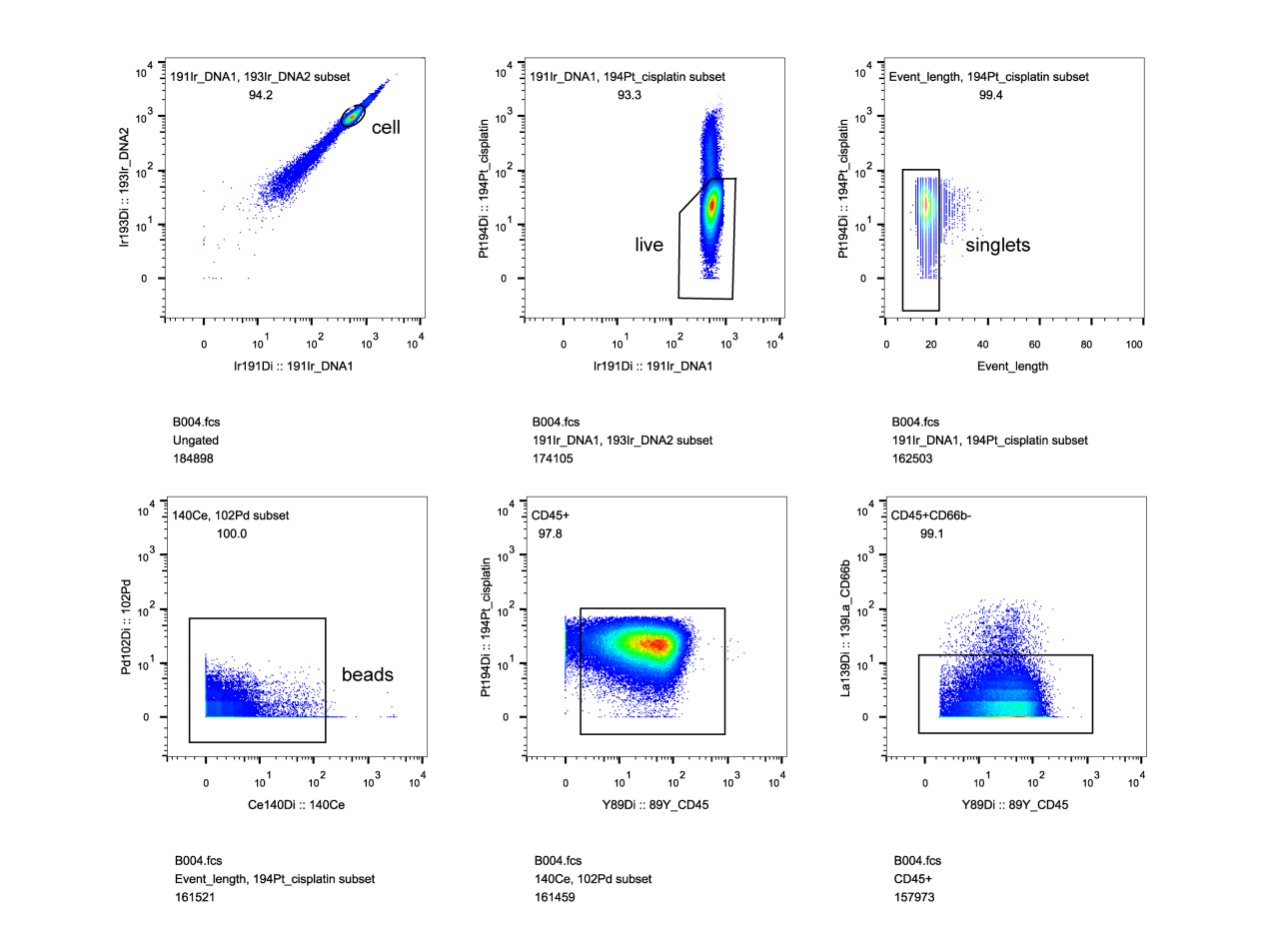


**Supplementary Figure 1 The gating strategy of the CyTOF.** The gating strategy to exclude debris, dead cells and doublets, leaving live, single immune cells.


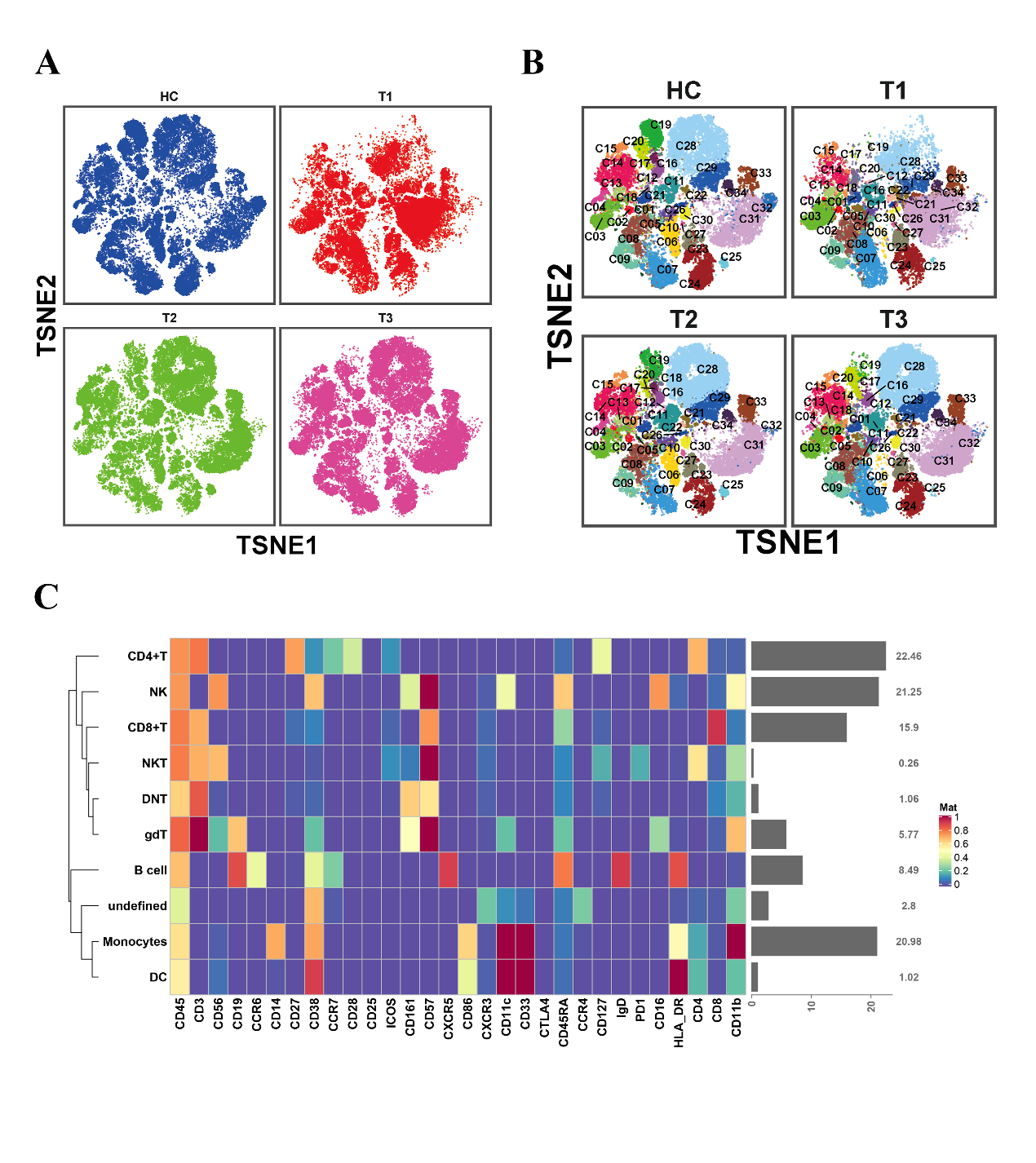


**Supplementary Figure 2 T-SNE maps and clustering analysis of major cell types.** (A) t-SNE maps of different groups. (B) t-SNE maps of all subpopulations in different groups. (C) Clustering of major cell types.


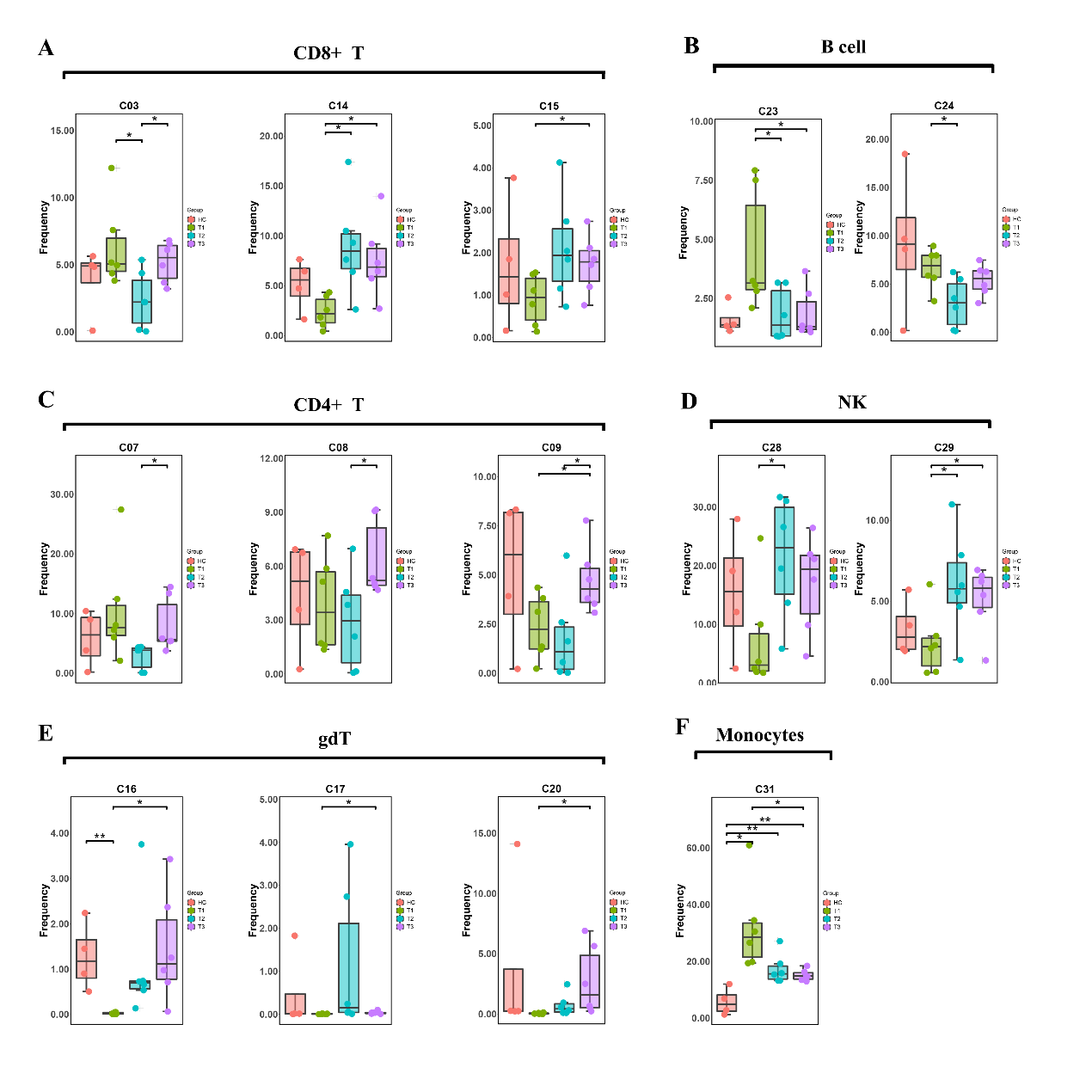


**Supplementary Figure 3 Subpopulations analysed by CyTOF that revealed significant changes using non-parametric Mann–Whitney U test**. The subpopulations of CD8+ T cells (A), B cells (B), CD4+ T cells (C), NK cells (D), gamma-delta T cells (E) and monocytes (F) revealed significant differences in comparison with the ratio among healthy controls and different time points.


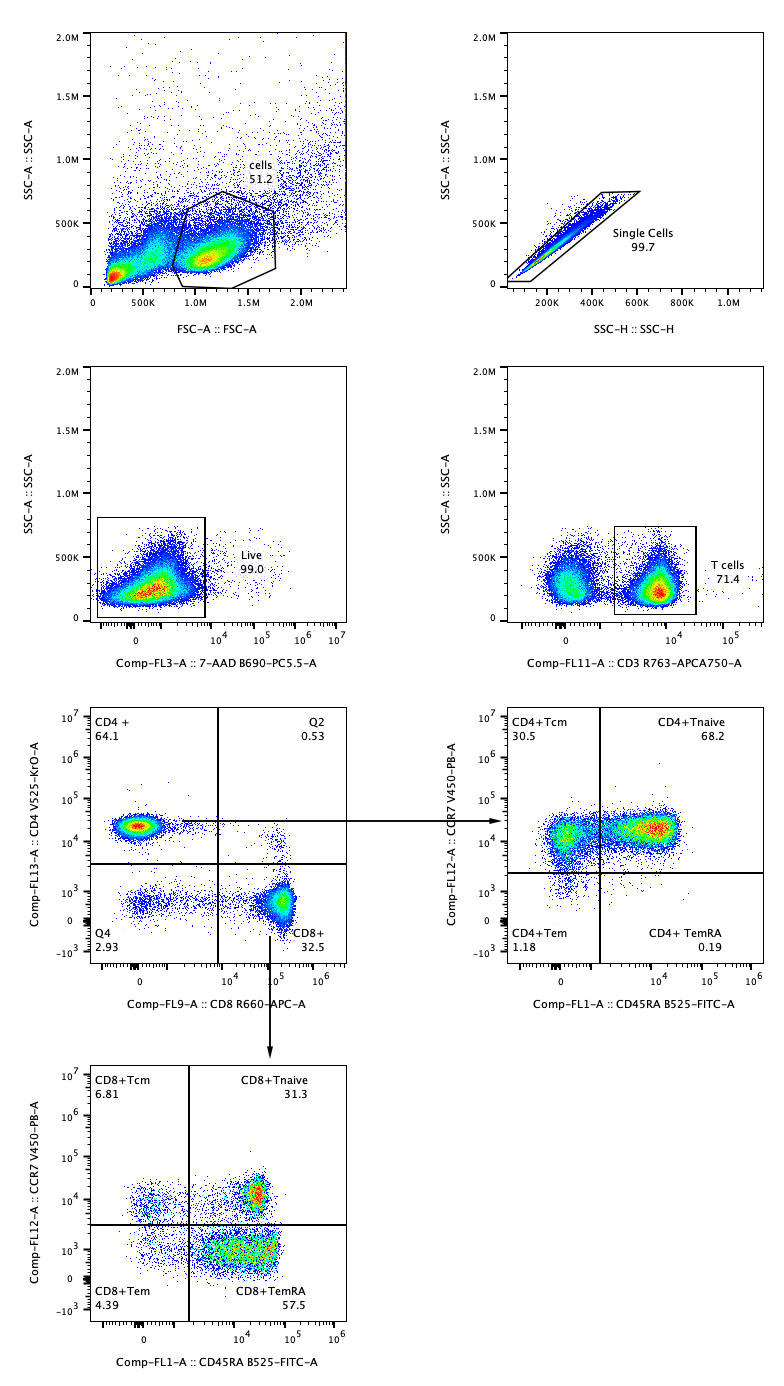


**Supplementary Figure 4 The gating strategy used to identify populations of CD4+ and CD8+ T cells.** Gating strategy to identify naïve T cells, TCM, TEM, TEMRA within CD8+ T cells and CD4+ T cells. Naïve T cells are identified as CCR7+CD45RA+; TCM are CCR7+CD45RA−;TEM are CCR7−CD45RA−; TEMRA are CCR7−CD45RA+.
